# Supplementary material for: Unlocking insights from complex data: Leveraging heat maps for decision-making in LMIC
Source: PLoS One. 2025 Sep 26;20(9):e0332394. doi: 10.1371/journal.pone.0332394 (PMC12468749; doi:10.1371/journal.pone.0332394)

**S1 Figure: Moran’s I for Condom’s Contraceptive**


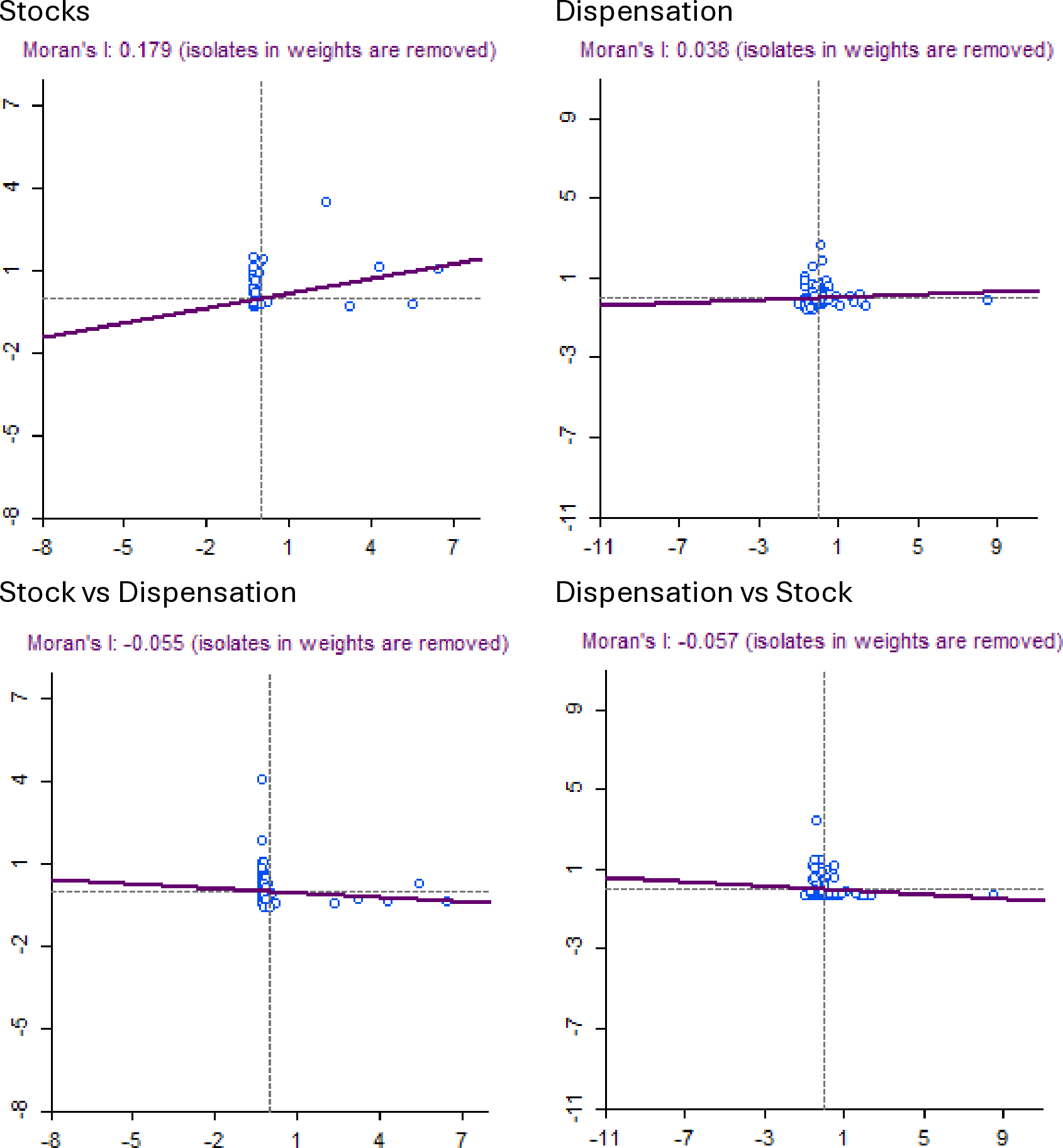


**S2 Figure: Moran’s I for Injection Contraceptive**


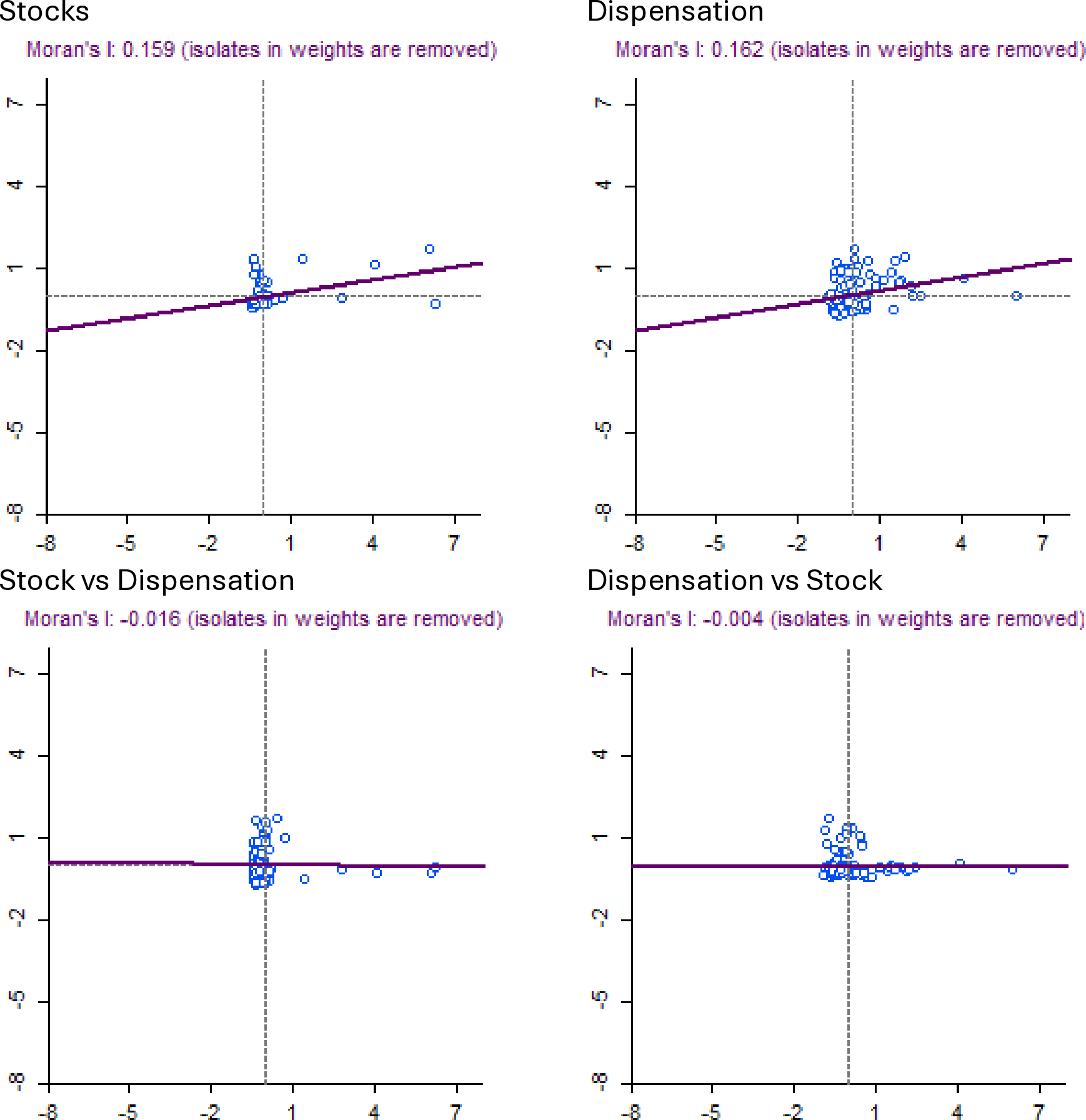


**S3 Figure: Moran’s I for IUDs Contraceptive**


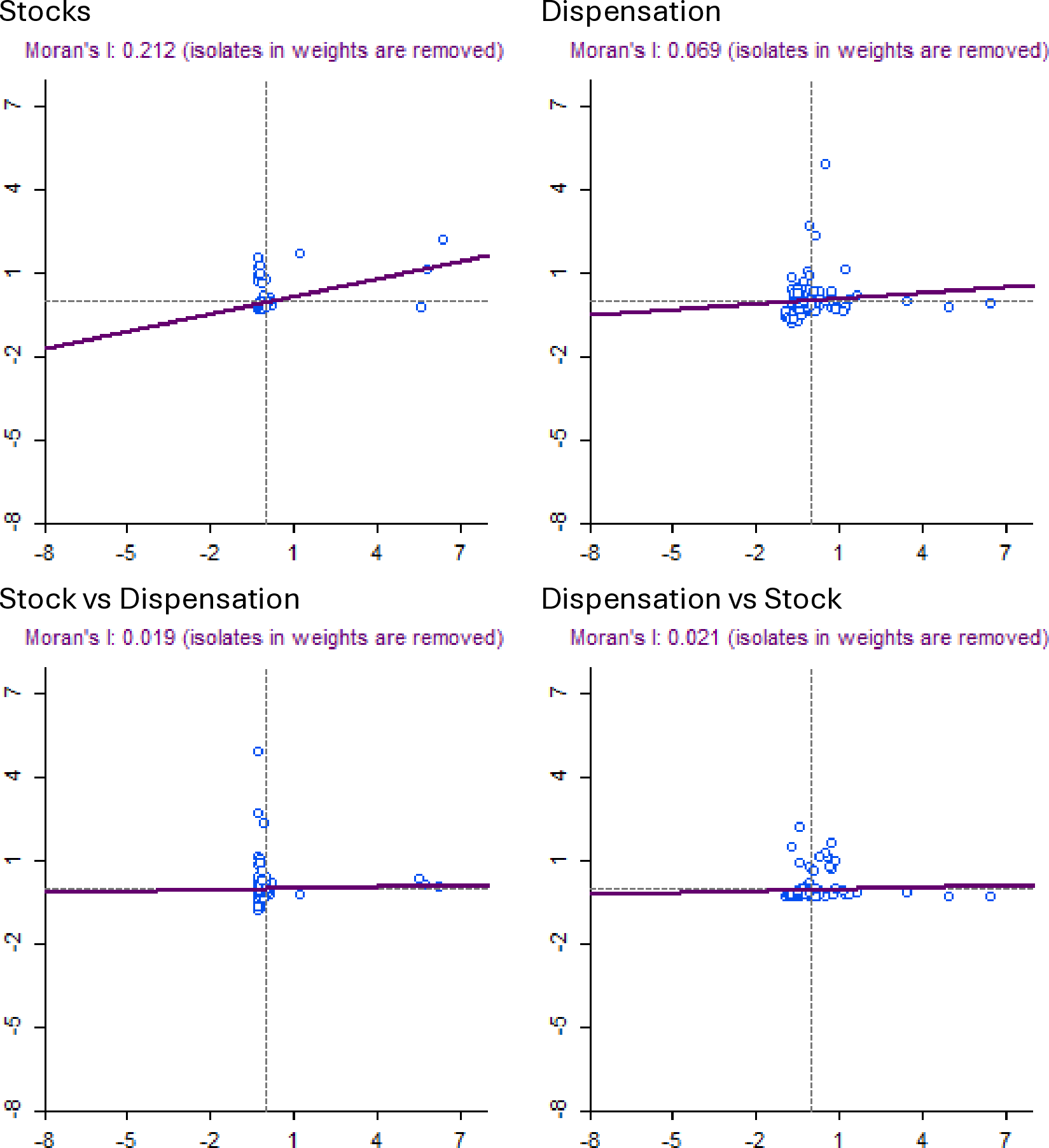

Supplement: S1 Appendix — (DOCX) [file pone.0332394.s001.docx]
