# Supplementary material for: Unlocking insights from complex data: Leveraging heat maps for decision-making in LMIC
Source: PLoS One. 2025 Sep 26;20(9):e0332394. doi: 10.1371/journal.pone.0332394 (PMC12468749; doi:10.1371/journal.pone.0332394)

**S4 Figure: Cluster and Significance Maps for Condom Contraceptive**


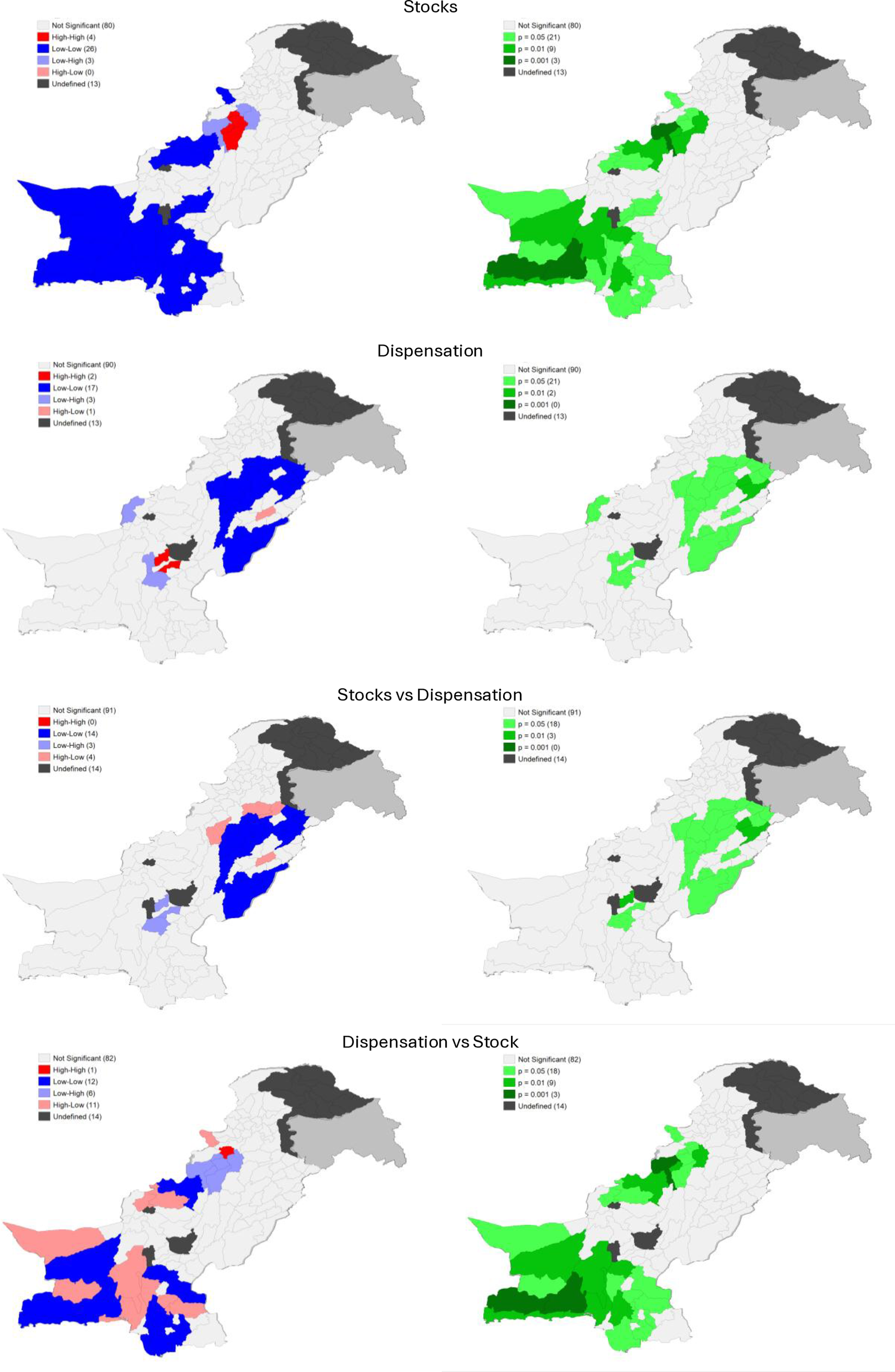


**S5 Figure: Cluster and Significance Maps for Injection Contraceptive**


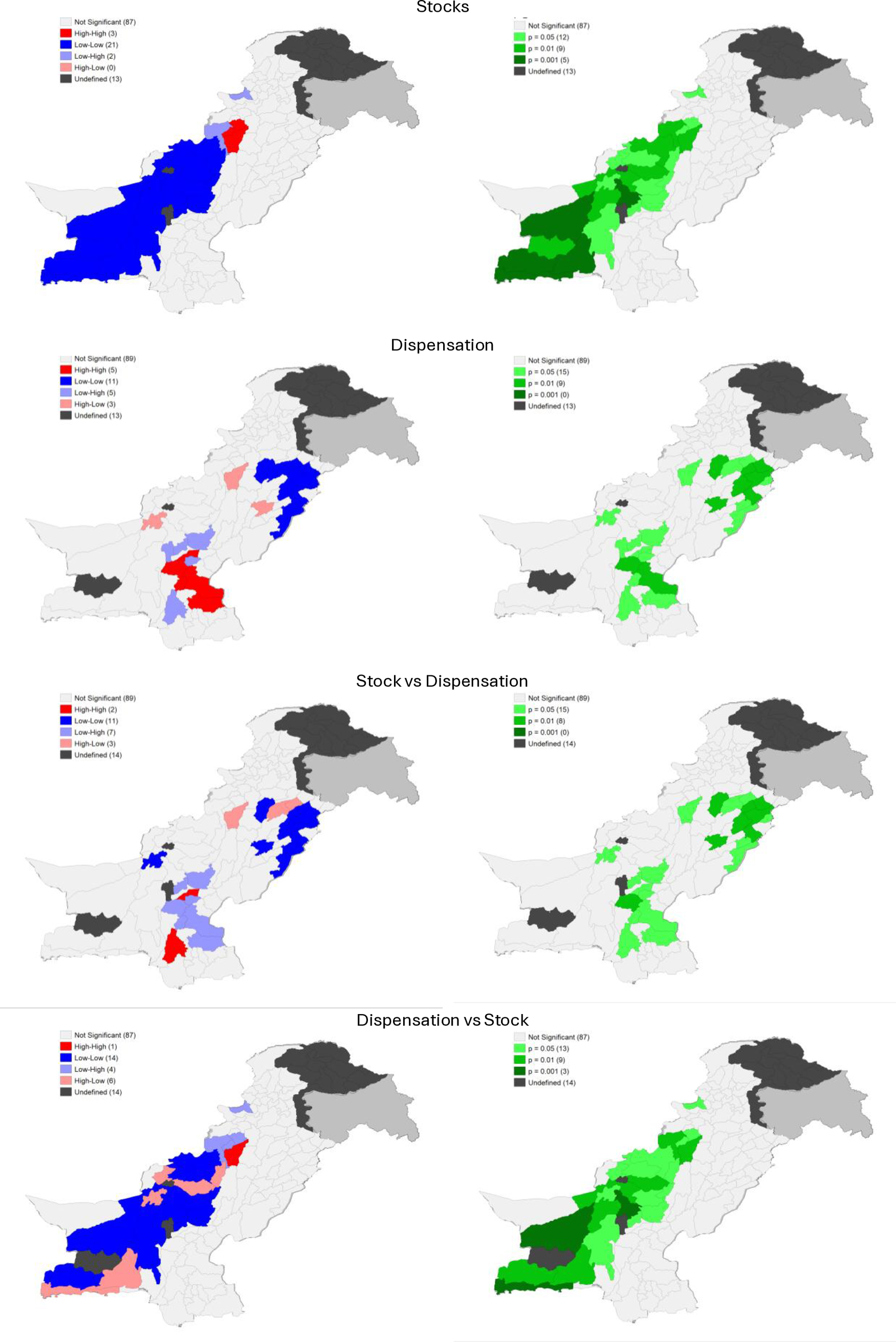


**S6 Figure: Cluster and Significance Maps for IUDs Contraceptive**


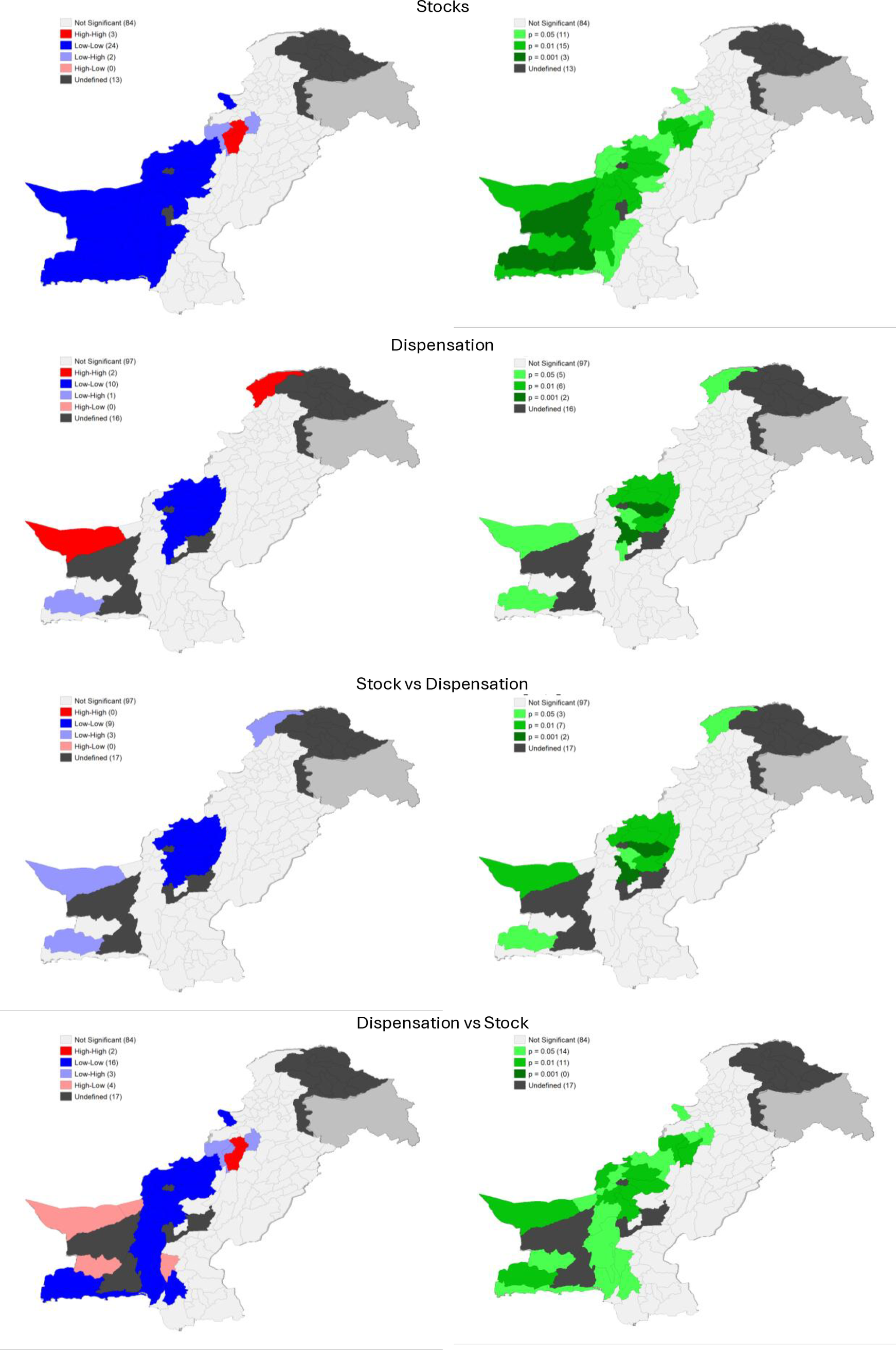

Supplement: S2 Appendix — (DOCX) [file pone.0332394.s002.docx]
