# Supplementary material for: Unlocking insights from complex data: Leveraging heat maps for decision-making in LMIC
Source: PLoS One. 2025 Sep 26;20(9):e0332394. doi: 10.1371/journal.pone.0332394 (PMC12468749; doi:10.1371/journal.pone.0332394)

PLOS ONE

Unlocking Insights from Complex Data: Leveraging Heat Maps for Decision-Making in LMIC

--Manuscript Draft--

**Manuscript Number:**	PONE-D-25-06311
**Article Type:**	Research Article
**Full Title:**	Unlocking Insights from Complex Data: Leveraging Heat Maps for Decision-Making in LMIC
**Short Title:**	Unlocking Insights from Complex Data
**Corresponding Author:**	Adnan Ahmad Khan<br>Research and Development Solutions<br>Islamabad, ICT PAKISTAN
**Keywords:**	spatial heat maps, contraceptives, vaccines, health logistics, low- and middle-income countries, Pakistan, data-driven decision-making, temporal mapping, public health policy
**Abstract:**	Introduction: In low- and middle-income countries (LMICs), health outcomes are often constrained by inadequate or misdirected resource allocation and limited access to services such as contraception and immunization. We explore the use of spatial heat maps to analyze the distribution of contraceptives and vaccines in Pakistan and to depict these analyses as visualizations.<br>Methods: We used data from national contraceptive (cLMIS) and vaccine logistics management information systems (vLMIS). We applied univariate, bi-variate, and tri-variate spatial heat maps to assess contraceptive and vaccine stock levels, consumption, and wastage across districts. For contraception, we standardized stocks per 100,000 married women of reproductive age (MWRA) and consumption rates. In immunization, we focused on Pentavalent-3 (Penta3) vaccine outreach, dropout rates, and Bacillus Calmette–Guérin (BCG) vaccine wastage.<br>Results: Temporal and spatial trends highlighted regional disparities, revealing that developed regions like Punjab had better stock availability, while underserved areas like Balochistan faced higher consumption rates and stockouts. We also show the effect of inputs (supplies, outreach) on rate of utilization of services (contraceptive uptake and vaccine coverage). Finally we depict how these visualizations can help track changes in programming over time.<br>Conclusions: Our findings show that integrating spatial data visualization with health logistics data identifies critical gaps in health service supply and demand, guiding policymakers in resource allocation, stock management, and service outreach. This scalable approach suits systems with limited analytical resources, as many analyses can be automated and embedded in datasets, providing policymakers with a focused set of visualizations for interpretation, avoiding the need for extensive training or deploying analysis teams at local levels. By leveraging spatial and temporal data, this method supports efficient health system strengthening and resource allocation in LMIC.
**Order of Authors:**	Muhammad Ibrahim, MS Economics
	Olan Naz, MS Economics
	Amal Fatima Mohiuddin
	Habib Ur Rehman
	Adnan Ahmad Khan, MBBS, MS
**Additional Information:**	
**Question**	**Response**
**Financial Disclosure**<br><br>Enter a financial disclosure statement that	Yes

Powered by Editorial Manager® and ProduXion Manager® from Aries Systems Corporation

describes the sources of funding for the work included in this submission. Review the submission guidelines for detailed requirements. View published research articles from *PLOS ONE* for specific examples.<br><br>This statement is required for submission and **will appear in the published article** if the submission is accepted. Please make sure it is accurate.<br><br>**Funded studies**<br>Enter a statement with the following details:<br>• Initials of the authors who received each award<br>• Grant numbers awarded to each author<br>• The full name of each funder<br>• URL of each funder website<br>• Did the sponsors or funders play any role in the study design, data collection and analysis, decision to publish, or preparation of the manuscript?<br><br>Did you receive funding for this work?	
Please add funding details.<br>as follow-up to "**Financial Disclosure**<br><br>Enter a financial disclosure statement that describes the sources of funding for the work included in this submission. Review the submission guidelines for detailed requirements. View published research articles from *PLOS ONE* for specific examples.<br><br>This statement is required for submission and **will appear in the published article** if the submission is accepted. Please make sure it is accurate.	This work has been funded by the Bill & Melinda Gates Foundation (INV 051108). The funding agency had no role in the development of this manuscript.

**Funded studies**<br>Enter a statement with the following details:<br>• Initials of the authors who received each award<br>• Grant numbers awarded to each author<br>• The full name of each funder<br>• URL of each funder website<br>• Did the sponsors or funders play any role in the study design, data collection and analysis, decision to publish, or preparation of the manuscript?<br><br>Did you receive funding for this work?"	
Please select the country of your main research funder (please select carefully as in some cases this is used in fee calculation).<br>as follow-up to "**Financial Disclosure**<br><br>Enter a financial disclosure statement that describes the sources of funding for the work included in this submission. Review the submission guidelines for detailed requirements. View published research articles from *PLOS ONE* for specific examples.<br><br>This statement is required for submission and **will appear in the published article** if the submission is accepted. Please make sure it is accurate.<br><br>**Funded studies**<br>Enter a statement with the following details:<br>• Initials of the authors who received each award<br>• Grant numbers awarded to each author<br>• The full name of each funder<br>• URL of each funder website<br>• Did the sponsors or funders play any role in the study design, data collection and analysis, decision to publish, or preparation of the manuscript?<br><br>Did you receive funding for this work?"	UNITED STATES - US
**Competing Interests**	The authors have declared that no competing interests exist.

Use the instructions below to enter a competing interest statement for this submission. On behalf of all authors, disclose any competing interests that could be perceived to bias this work—acknowledging all financial support and any other relevant financial or non-financial competing interests.<br><br>This statement is required for submission and **will appear in the published article** if the submission is accepted. Please make sure it is accurate and that any funding sources listed in your Funding Information later in the submission form are also declared in your Financial Disclosure statement.<br><br>View published research articles from *PLOS ONE* for specific examples.<br><br>**NO authors have competing interests**<br><br>Enter: *The authors have declared that no competing interests exist.*<br><br>**Authors with competing interests**<br><br>Enter competing interest details beginning with this statement:<br><br>*I have read the journal's policy and the authors of this manuscript have the following competing interests: [insert competing interests here]*<br><br>* typeset	
**Ethics Statement**<br><br>Enter an ethics statement for this submission. This statement is required if the study involved:<br><br>• Human participants<br>• Human specimens or tissue<br>• Vertebrate animals or cephalopods<br>• Vertebrate embryos or tissues	N/A

Powered by Editorial Manager® and ProduXion Manager® from Aries Systems Corporation

• Field research<br><br>Write "N/A" if the submission does not require an ethics statement.<br><br>General guidance is provided below. Consult the submission guidelines for detailed instructions. **Make sure that all information entered here is included in the Methods section of the manuscript.**	

**Format for specific study types**<br><br>**Human Subject Research (involving human participants and/or tissue)**<br>• Give the name of the institutional review board or ethics committee that approved the study<br>• Include the approval number and/or a statement indicating approval of this research<br>• Indicate the form of consent obtained (written/oral) or the reason that consent was not obtained (e.g. the data were analyzed anonymously)<br><br>**Animal Research (involving vertebrate animals, embryos or tissues)**<br>• Provide the name of the Institutional Animal Care and Use Committee (IACUC) or other relevant ethics board that reviewed the study protocol, and indicate whether they approved this research or granted a formal waiver of ethical approval<br>• Include an approval number if one was obtained<br>• If the study involved *non-human primates*, add *additional details* about animal welfare and steps taken to ameliorate suffering<br>• If anesthesia, euthanasia, or any kind of animal sacrifice is part of the study, include briefly which substances and/or methods were applied<br><br>**Field Research**<br><br>Include the following details if this study involves the collection of plant, animal, or other materials from a natural setting:<br>• Field permit number<br>• Name of the institution or relevant body that granted permission	
**Data Availability**<br><br>Authors are required to make all data underlying the findings described fully available, without restriction, and from the time of publication. PLOS allows rare exceptions to address legal and ethical concerns. See the PLOS Data Policy and FAQ for detailed information.	No - some restrictions will apply

Powered by Editorial Manager® and ProduXion Manager® from Aries Systems Corporation

A Data Availability Statement describing where the data can be found is required at submission. Your answers to this question constitute the Data Availability Statement and **will be published in the article**, if accepted.<br><br>**Important:** Stating 'data available on request from the author' is not sufficient. If your data are only available upon request, select 'No' for the first question and explain your exceptional situation in the text box.<br><br>Do the authors confirm that all data underlying the findings described in their manuscript are fully available without restriction?	
**Describe where the data may be found in full sentences. If you are copying our sample text, replace any instances of XXX with the appropriate details.**<br><br>• If the data are **held or will be held in a public repository**, include URLs, accession numbers or DOIs. If this information will only be available after acceptance, indicate this by ticking the box below. For example: *All XXX files are available from the XXX database (accession number(s) XXX, XXX.).*<br>• If the data are all contained **within the manuscript and/or Supporting Information files**, enter the following: *All relevant data are within the manuscript and its Supporting Information files.*<br>• If neither of these applies but you are able to provide **details of access elsewhere**, with or without limitations, please do so. For example:<br><br>*Data cannot be shared publicly because of [XXX]. Data are available from the XXX Institutional Data Access / Ethics Committee (contact via XXX) for researchers who meet the criteria for access to confidential data.*<br><br>*The data underlying the results presented in the study are available from (include the name of the third party*	The data has been acquired from "The Chemonics" under collaboration with Research and Development Solutions (RADS), Islamabad, Pakistan. However, the data will be available upon request.

and contact information or URL).<br>• This text is appropriate if the data are owned by a third party and authors do not have permission to share the data.<br><br>* typeset	
Additional data availability information:	

Manuscript Click here to access/download;Manuscript;Manuscript.docx

Unlocking Insights from Complex Data: Leveraging Heat Maps for Decision-Making in LMIC

Muhammad Ibrahim[1], Olan Naz[1], Amal Fatima Mohiuddin[1], Habib Ur Rehman[1], Adnan Ahmad Khan[1,2*]

[1] Research and Development Solutions (RADS), Islamabad, Pakistan

[2] Ministry of National Health Services, Regulations and Coordination (MoNHSRC), Islamabad, Pakistan

*Corresponding Author

Adnan Ahmad Khan
Research and Development Solutions
Roshan Aaj Center, Asad Market, I-9/4
Islamabad, Pakistan 44000
Email: adnan@resdev.org

Introduction

Access to effective services is a major to family planning in Pakistan and accounts for over 70% of non-use by those wishing to use FP [1]. A key part of access to effective contraception is the availability of contraceptives at facilities where couples visit. Similarly, inadequate supplies have been a significant constraint in ensuring high vaccine coverage for preventable childhood illnesses. In ability to ensure services and supplies in a timely manner leads to missed opportunities in family planning or other preventive or promotive services such as routine vaccination and exacerbate health outcomes and social disparities. While policy makers seek to address these gaps, it is imperative that their choices are guided by evidence, such how services and supplies are managed and taken up and where are the gaps. Effective utilization of such data can help identify where services and supplies are needed the most, thereby improving health and well-being.

While considerable data are available from healthcare systems, surveys, and various channels, their potential for informed policymaking remains largely untapped [2],due to limited capacity to harness, manage and interpret data, time and consistently [3].Often, this interpretation would include triangulation of disparate data sources, analysis, and depiction into forms, such as visualization to make data more accessible for decision makers who may not be data experts [4-7]. Additionally, information such as population and program data change rapidly, and interpretations must keep pace with these changes to stay relevant. This underscores the need to utilize adaptable visualization methods that can be replicated routine and frequently to facilitate real-time interpretations [6, 8, 9].

Basic bar graphs and pie charts are often the mainstay of presentations but convey limited information. By contrast, spatial heat maps offer greater flexibility and enhanced readability, effectively illustrating regional variations, highlighting hotspots or time trends [10]. One common advantage of spatial heat maps is that the surface area of country, province or district is always presented through the coordinates present. In addition to depicting simplified indicators, such as stock levels, total consumption, or facility counts, these maps can also simplify more complex visualizations such as the intersection of multi-indicators.

Decision-making is a cognitive process where a person evaluates factors to solve a problem [11], depending on the complexity of the decision (such as the number of criteria and outcomes involved) [12]. While simpler decisions work with simple heuristics, complex ones require solutions that include ranking the quality and utility of data based on predetermined criteria [13]. Limited guidelines illustrate how to design indicators to support complex reasoning across different contexts [14]. Visualizations utilize the brain's sensory capacity to depict complex relationships between data points [15], such as simplifying the relationships of indicators not just across spatial dimensions, but also relate related them over time and with other variables [16, 17].

We present one such application in the form of matching supply and demand (two or three factors combined) for contraceptives and vaccines across all districts of Pakistan through spatial heat maps, using data available from the national contraceptive and vaccine logistics management information system (cLMIS and vLMIS). In fact, this technique can be applied to other essential medicines, availability of human resources for health, optimizing various services across or within districts and more. If data are available, the technique can help disaggregate such depictions at more granular levels such as union councils (the smallest administrative units in Pakistan) or individual facilities.

Data and Methods

Data from the cLMIS (contraceptives) and vLMIS (vaccines) portals provided by The Chemonics, through their work with the USAID Global Health Supply Chain Program-Procurement and Supply Management project in Pakistan. Stocks and consumption data for contraceptives were extracted, while vaccine data included indicators such as live birth targets (from now on referred as targeted population), stocks, vaccinations, and wastage. The supply chain for both contraceptives and vaccines is tracked by The Chemonics team from the central warehouse to provincial warehouses, district warehouses, and finally to individual facilities. The focus was on the final stage of supply where stocks are utilized by consumers. Data were aggregated at the district level to create spatial heat maps for broad discussion, which can be replicated at a granular level by district leadership. Monthly data for the calendar year 2022 were aggregated to provide annual estimates for both contraceptives and vaccines, except for one visual used to capture monthly disparities. All spatial heat maps are made using STATA 17.0.

Contraceptives data, comprising only two main variables—stocks and consumption—required careful assessment. Simply displaying these variables on a spatial heat map is insufficient, as it fails to provide meaningful insights. Districts with higher populations naturally require more stock, and consumption alone does not indicate whether the available stocks are being appropriately utilized. For instance, if a district has higher stocks relative to others but a smaller population, and the consumption is below 20%, the information can be misleading. A decision-maker might incorrectly conclude that Married Women of Reproductive Age (MWRA) are not using enough contraceptives, whereas the issue could be overstocking. Therefore, it is essential to standardize these variables for a clearer understanding of the data and look at both indicators together.

To standardize the stocks, the available stock per district was divided by the number of MWRA in a district and multiplied by 100,000 for better understanding of estimates. MWRA numbers were obtained from the Population Census 2017, resulting in the variable “Stocks available per 100,000 MWRA per district” formula shown in Table 1. After 2017, several new districts were added to the cLMIS data which were present in the population census data. Therefore, in the cLMIS data, we aggregated the values for the following districts: Sibi and Lehri, Chitral Lower and Chitral Upper, Kohistan Upper and Kohistan Lower, Duki and Loralai, Killa Abdullah and Chaman, and Shaheed Sikandar Abad and Kalat. Furthermore, cLMIS categorized Karachi as a single entity along with the seven districts within Karachi city, as a result, we aggregated all the data of Karachi city. These values were then categorized into five quantiles. Consumption was standardized by calculating the percentage of available stock consumed per district. This was done by dividing the consumption by the available stock, resulting in four categories: up to 25% of stock consumed (very poor consumption pattern), 25-50% consumed (relatively poor), 50-75% consumed (relatively better), and above 75% consumed. Separately, these spatial heat maps provide limited information, but when analyzed together, they offer more comprehensive insights for targeted policy development. These results are shown in Figure 1 using data for condom contraceptives.

Initially, two separate spatial maps were created to demonstrate how both indicators can be read, although they may be confusing to interpret together. To provide clearer insights, a bi-variate spatial heat map was developed. On the y-axis, stocks per 100,000 MWRA are shown, now divided into four quartiles, with lighter shades indicating higher availability. On the x-axis, color variations represent changes in stock consumption as previously discussed (up to 25%, 25-50%, and so on). This bi-variate structure allows for the examination of sixteen distinct data points, as illustrated in results section (Figure 3). The bi-variate spatial heat maps for condoms,

injections, and intrauterine devices (IUDs) are analyzed both separately and all three together. Condoms, being a one-time use contraceptive, show different stock and consumption patterns compared to injections, which provide contraception for one to three months, and IUDs, which last five to eight years. Comparing these three bi-variate spatial maps reveals varying preferences and usage patterns among MWRA.

With the utilization of bi-variate spatial maps established for interconnected indicators, their application is extended to immunization indicators. The focus is on Sindh province, as the Chemonics team validated the data on their portal for this region for vaccinations. There is a notable link between outreach vaccination services and vaccination dropout rates in Pakistan [18, 19]. The Penta3 vaccine is crucial in protecting children against diphtheria, tetanus, pertussis (whooping cough), hepatitis B, and Haemophilus influenzae type b (Hib). Pentavalent third dose (Penta3) outreach vaccination outcomes are analyzed with Penta3 dropout rates. Calculations of indicators are shown in Table 1. A dropout rate refers to the percentage of children who received the first dose of the vaccine but did not complete the third dose. Monthly graphs were created to display Penta3 doses administered through outreach against Penta3 dropout rates. Demonstrating monthly bi-variate maps reveal significant spatial and temporal variations pertinent to vaccination coverage.

Lastly, since vaccines differ from contraceptives, tri-variate spatial maps were developed to assess vaccine data, incorporating stock availability, consumption, and wastage. Data was taken of Bacillus Calmette-Guerin (BCG) vaccine for 2022 across Sindh. Vaccine wastage is a crucial indicator that complements consumption patterns, [20] referred to as vaccinations. A new visualization strategy was employed to avoid confusion from using multiple colors. This approach integrates information from three variables in an easy-to-understand form. The base color on the spatial map represents stock availability per targeted population. Circles of

varying size and color (red indicating low, green indicating adequate) depict vaccination rates per targeted population in each district. Additionally, pie charts overlaid on the circles represent wastage as a percentage of the available stock per district.

The cut-off points for the legend were determined through the quartile's method, except for the consumed proportion of stocks available where we used percentages as the cutoff point and the wastage rate is a continuous graph starting from 0 to 100%. The cutoff points are shown in the table below (Table 1).

Table 1: Indicators for Spatial Heat Maps per District

Sr. No.	Indicator (1)	Formula (2)	Cut-off points of Data in Spatial Maps (3)	Commodity/Vaccine (Illustrated in Figure) (4)
1	Stocks per 100,000 MWRA	$\left(\frac{Total\ Stocks}{MWRA}\right) \times 100{,}000$	0-43,000, 43,000-122,000, 122,000-296,000, >296,000	Condoms (3)
			0-5,000, 5,000-13,500, 13,500-21,000, >21,000	Injections (3)
			0-1,200, 1,200-3,000, 3,000-6,000, >6,000	IUDs (3)
2	Consumption Proportion of Stocks Available	$\left(\frac{Total\ Consumption}{Total\ Stocks}\right) \times 100$	0-25%, 25-50%, 50-75%, 75-100%	Condoms, Injections & IUDs (2 & 3)
3	Pentavalent-3 outreach per facility	$\left(\frac{Vaccinations\ through\ outreach}{No.of\ Facilities}\right)$	0-25, 25-50, 50-75, >75	Pentavalent 3 (4)
4	Pentavalent-3 Dropout Rates	$\left(\frac{Penta1 - Penta3}{Penta1}\right) \times 100$	<-50%, -50-0%, 0-50%, >50%	Pentavalent 3 (4)
5	Stocks per 1,000 targeted population	$\left(\frac{Total\ Stocks}{Live\ Birth\ Targets}\right) \times 1{,}000$	1,600-1,850, 1,850-2,000, 2,000-2,300, >2,300	BCG (5)
6	Vaccinations per 1,000 targeted population	$\left(\frac{Total\ Vaccinations}{Live\ Birth\ Targets}\right) \times 1{,}000$	800-930, 930-1,060, 1,060-1,190,	BCG (5)

			>1,190	
7	Proportion of Wastage per Available Stock	$\left(\frac{Total\ Wastage}{Total\ Stocks\ Available}\right) \times 100$	0-100%	BCG (5)

Note: Above table doesn't show the cut off points for the univariate spatial maps created for contraceptives, as the values are visible on the legend.

Results

Distribution and consumption of condoms are used to illustrate the considerable regional variation across Pakistan. Figure 1 describes these in terms of absolute numbers. Central/ eastern regions, particularly Punjab, have the highest stock levels, sometimes exceeding 2.5 million condoms in some districts while in south (parts of Sindh), west (Balochistan) and the northwest (Khyber Pakhtunkhwa – KP) have lower stock levels, ranging from 55-705 thousand condoms per district. Central and northeastern areas display moderate to high stocks, ranging from 0.7 to over 2.5 million. Consumption patterns are similar. The same areas that have the lowest stocks also have the lowest consumption, while those with the highest stocks have the most consumption.

Figure 1: Stocks and Consumption of Condoms in Actual Quantities

However, simple depictions of quantity don't account for the need for such commodities. For this we adjusted the quantities for the population and depicted quantities per 100,000 Married Women of Reproductive Age (MWRA) (Figure 2). The resulting picture (Figure 2) remains similar for stocks in that, areas that had the least stocks overall also have the lowest stocks when adjected for population. However, the consumption patterns change dramatically. Areas in southwest (Balochistan), south (parts of Sindh) and northwest (parts of KP) that had the least stocks also have the most of their stocks consumed, resulting in stockouts. In the east (most of Punjab), where the more stock was supplied, relatively lesser proportion of the stock was consumed, suggesting oversupply of condoms relative to the population-based need.

Figure 2: Stocks and Consumption of Condoms Adjusted for Population

Bi-variate Spatial Maps and importance of its interpretation

This increasing level of complexity from matching supplies and consumption can be depicted in a single figure to give decision makers one composite figure to understand the situation. Figure 3 shows the layering of stocks and consumption together to show the interaction between them. The Y axis shows stocks, and the X axis shows consumption. The figure for condoms confirms that districts in the south, southwest and northeast that have the least supplies also have the least consumption. While in central and eastern districts there are higher stocks and relatively low consumption.

The pattern for contraceptive injections is very similar to condoms. However, the situation is very different for IUCD. There is the least stocking and consumption in Balochistan

(southwest). Sindh (south) sees moderate to high stocks but low consumption while Punjab (central and east) has the highest supplies and moderate consumption.

Figure 3: Regional Disparities in Contraceptive Availability and Consumption in Pakistan

Spatial and Temporal Insights using Bi-variate Maps

Another layer of information can be incorporated to inform about the effect of time. Figure 4 shows monthly changes in the rate of dropouts for the Penta-3 vaccine as a factor of community outreach across various districts of the Sindh province. There are relatively high dropout rates in many southern and central districts in the first quarter, with some improvements by March. Tharparkar, Sujawal, Dadu and Khairpur consistently show high dropout rates throughout the quarter. Sukkur and Ghotki (in the north) show gradual improvements with relatively lower dropout rates and increasing outreach by the end of the quarter, particularly in March. These patterns can inform about seasonal variations or changes in the level of implementation of interventions over time.

Figure 4: Spatial and Temporal Trends in Outreach per Facility and Penta-3 Dropout Rates in Sindh

Deeper Insights using Three Indicators in Tri-variate Spatial Map

In addition to the temporal changes the tri-variate spatial map also allows adding a third dimension. For e.g., Sindh province data is displayed for vaccine stock and consumption (both per 1,000 targeted population) vs. the wastage shown as proportion of available stock across districts (Figure 5).

Figure shows that southern (Sujawal, East Karachi, Korangi, Malir and Matiari) and central (Larkana and Jacobabad) districts have the highest stock levels per 1,000 targeted population, while some northern districts (Kambar Shahdad Kot, Naushahro Feroze) have lower stock levels. Overall, adjusted stock levels show that there are ample stocks available per district. However, vaccination and wastage rates differ across districts.

High vaccination rates are observed in northern and central districts of Sindh such as Jacobabad, Dadu, Kashmore and Shaheed Benazir Abad, whereas low vaccination rates in the south (Sujawal, Thatta, and all districts of Karachi), highlighting potential issues in either vaccine distribution or uptake. Significant vaccine wastage is observed, above the 50% threshold, in southern districts such as Sujawal, Hyderabad, and parts of Karachi, indicate inefficiencies in vaccine management. Lower vaccine wastage was observed in central and northern districts (Shaheed Benazir Abad, Naushahro Ferozeand, Shikarpur, and Ghotki). However, this must be interpreted along with the prior observation of low stocks, suggesting that these lower stocks are being consumed.

Figure 5: Geospatial Mapping of BCG Vaccine Distribution, Uptake, and Wastage in Sindh

Notes: Zoomed part (left of part of the Figure, below Legend) are the districts of Karachi City: South Karachi, West Karachi, Central Karachi, East Karachi, Korangi and Malir.

Discussion

Improving data systems and technology allowing increasing amounts and granularity of data to be collected about health programs also require better interpretation techniques. In addition as more data become available and there are fewer professionals that can interpret these data, particularly in LMIC, better techniques can make data and its interpretation accessible for decision makers. We present four such techniques.

Simple heat maps represent an improvement over basic tables, particularly when viewing data from large numbers of localities or categories, such as the over 150 districts in Pakistan. The next level improvement is to adjust stock amounts for the population in need. A further layer of complexity was to depict both consumption and supplies in the same figure, using bivariate graphs. Alternatively, changes in indicators over time can be viewed in side-by-side figures that track temporal trends. Finally, interaction of variables such as relationship between the level of outreach and immunization coverage can be explored using tri-variate figures.

Exploratory data techniques effectively support multicriteria spatial decision-making by enhancing the interaction between maps and data graphs/points [21]. The integration of spatial mapping and analysis techniques has enabled the use of an expanding array of routinely available geographic data sources for public health care, research and development [22]. Spatial methods in epidemiology clarify the distribution of population health and its local causes, guiding targeted interventions to prevent disease and promote health [23]. Additionally, recent studies show that spatial heat maps help identify the prevalence and spread of infectious diseases, such as malaria and COVID-19 [24-26]. Similarly, our research's spatial heat maps reveal significant regional disparities in the availability and consumption of essential resources across Pakistan, particularly in contraceptives and immunization supplies. This highlights district-level supply and demand issues through specific indicators and

underscores the need for resource redistribution across districts with higher stock levels and lower demand.

The analysis of these spatial disparities also facilitates targeted interventions, allowing decision-makers to assess neighboring districts and redistribute resources where needed. For instance, if one district has surplus stocks, these can be redirected to neighboring districts facing shortages. This integration of data helps decision-makers understand the underlying problems better and reduces cognitive complexity by focusing on key factors, thus streamlining the decision-making process. Furthermore, our assessment of temporal-spatial heat maps allows for analyzing patterns over time. For example, Sujawal district (Figure 4) serves as a key example of trend analysis; as outreach services decline, the dropout rate for Penta3 vaccinations increases at specific months. The data underscores the importance of continuous monitoring and adaptive strategies to improve immunization outcomes, particularly in regions with high dropout rates.

Analyzing spatial and temporal trends of the indicator enables us to track its sources within or across districts, while risk maps based on the latest data enhance intervention measures [24]. In Figure 5, a major supply issue is the wastage rate, which we incorporated using tri-variate spatial heat maps. Policymakers should address distribution inefficiencies in districts with high wastage and low vaccination rates, improve storage facilities in areas with high wastage, and enhance community outreach and educational campaigns in regions with low vaccination rates to boost uptake. Frequent use of these visualizations will help analyze large datasets, enabling stakeholders to identify and address potential shortfalls easily.

These visualization techniques provide powerful tools for policymakers, featuring user-friendly interfaces that require minimal technical expertise to generate specific visualizations. By offering detailed, district-level insights into stock levels, consumption, and wastage, these

visualizations facilitate data-driven decision-making, ultimately improving program efficiency and resource allocation to meet regional needs. Their scalability ensures a consistent framework for ongoing analysis and monitoring across different districts and over time. They enhance data clarity and accessibility, contributing to the continuous improvement of public health programs. Bivariate or trivariate spatial heat maps help identify areas that require stock adjustments, outreach service enhancements, and wastage reduction, providing valuable tools for policy formulation and implementation. This approach not only improves the efficiency of contraceptive and immunization programs but also ensures that resources are allocated appropriately to meet the needs of various regions.

Once decision-makers identify the concept and analysis syntax, they can embed analysis leading to such spatial heat maps directly into the database or data dashboards using short programs called Application Programming Interfaces (APIs)[27]. Furthermore, interactive maps can significantly enhance spatial decision-making [28] upon clicking the district decision can get statistical of that district. This enables decision-makers to generate them monthly or more frequently with a single click, eliminating the need for complex data analysis. Our district example can be replicated for the entire country, a single province, or even an individual facility. We can also extend the process to other commodities, such as medicines, supplies, vaccines, or even human resources for health. If we manage these commodities on the same dataset, the process simplifies by allowing users to click on filters from a drop-down menu [29].

Such tools facilitate analysis at both the macro and micro levels, helping decision-makers identify overstocked or understocked locations, understand demand patterns, and identify resources for redistribution between neighboring districts or facilities, leading to informed decisions. Our findings contribute to visualizations that allow users to query large datasets

with minimal human expertise, enabling better resource allocation at health facilities and ultimately improving health outcomes.

Conclusion

Our analysis of contraceptive and immunization data using bi-variate and tri-variate spatial heat maps reveals significant regional disparities in Pakistan. Urban centers, particularly in developed areas like Punjab and Sindh, show better stock availability and higher consumption rates than less developed regions such as Balochistan and southern Khyber Pakhtunkhwa. In immunization, our temporal and spatial analysis of Penta3 indicators highlights seasonal trends and persistent outreach challenges in southern Sindh. This necessitates focused and resource-intensive strategies to improve vaccination coverage. Addressing vaccine wastage through improved distribution and storage, along with enhanced community outreach, is crucial.

Integrating data point and spatial maps enhances decision-making processes. These visualizations offer detailed insights into resource distribution, facilitating data-driven decisions that optimize program efficiency. Spatial maps simplify the generation of actionable insights, empowering policymakers to make informed decisions quickly. Ultimately, spatial maps serve as essential tools for guiding health interventions, ensuring effective resource allocation to meet diverse regional needs. This research highlights the critical role of spatial analysis in improving public health outcomes through targeted policy actions.

Acknowledgment:

We would like to extend our gratitude to CHEMONICS Pakistan for providing the valuable data used in this study. Their contribution has been instrumental in enabling our research and enhancing our understanding of the supply and consumption dynamics of contraceptives in Pakistan.

References

1. (NIPS), N.I.o.P.S., *PAKISTAN DEMOGRAPHIC AND HEALTH SURVEY 2017-18*. 2018.
2. Roberts, J., et al., *From Ill-Defined Problems to Informed Decisions*.
3. Aung, T., et al., *Optimizing data visualization for reproductive, maternal, newborn, child health, and nutrition (RMNCH&N) policymaking: data visualization preferences and interpretation capacity among decision-makers in Tanzania.* Global Health Research and Policy, 2019. 4: p. 1-14.
4. Aung, T., D. Niyeha, and R. Heidkamp, *Leveraging data visualization to improve the use of data for global health decision-making.* Journal of global health, 2019. 9(2).
5. Zakkar, M. and K. Sedig, *Interactive visualization of public health indicators to support policymaking: An exploratory study.* Online journal of public health informatics, 2017. 9(2).
6. Raghupathi, W. and V. Raghupathi, *An empirical study of chronic diseases in the United States: a visual analytics approach to public health.* International journal of environmental research and public health, 2018. 15(3): p. 431.
7. Sadiku, M., et al., *Data visualization.* International Journal of Engineering Research And Advanced Technology (IJERAT), 2016. 2(12): p. 11-16.
8. Lavrač, N., et al., *Data mining and visualization for decision support and modeling of public health-care resources.* Journal of biomedical informatics, 2007. 40(4): p. 438-447.
9. Preim, B. and K. Lawonn. *A survey of visual analytics for public health*. in *Computer Graphics Forum*. 2020. Wiley Online Library.
10. Słomska-Przech, K., T. Panecki, and W. Pokojski, *Heat maps: Perfect maps for quick reading? comparing usability of heat maps with different levels of generalization.* ISPRS International Journal of Geo-Information, 2021. 10(8): p. 562.
11. Wang, Y. and G. Ruhe, *The cognitive process of decision making.* International Journal of Cognitive Informatics and Natural Intelligence (IJCINI), 2007. 1(2): p. 73-85.
12. Jelokhani-Niaraki, M. and J. Malczewski, *Decision complexity and consensus in Web-based spatial decision making: A case study of site selection problem using GIS and multicriteria analysis.* Cities, 2015. 45: p. 60-70.
13. Einhorn, H.J. and R.M. Hogarth, *Rationality and the sanctity of competence.* Behavioral and Brain Sciences, 1981. 4(3): p. 334-335.
14. MacEachren, A.M. *Distributed cognition: A conceptual framework for understanding map-based reasoning*. in *Proceeding of the 27th International Cartographic Conference (August 2015), International Cartographic Association. http://icaci.org/files/documents/ICC proceedings/ICC2015/papers/2/940. html*. 2015.
15. Roth, R.E. and A.M. MacEachren, *Geovisual analytics and the science of interaction: an empirical interaction study.* Cartography and Geographic Information Science, 2016. 43(1): p. 30-54.
16. Jankowski, P. and L. Richard, *Integration of GIS-based suitability analysis and multicriteria evaluation in a spatial decision support system for route selection.* Environment and Planning B: Planning and Design, 1994. 21(3): p. 323-340.
17. Leung, Y., *Intelligent spatial decision support systems*. 2012: Springer Science & Business Media.
18. Shiferie, F., et al., *Vaccination dropout and wealth related inequality among children aged 12–35 months in remote and underserved settings of Ethiopia: a cross-sectional evaluation survey.* Frontiers in Pediatrics, 2023. 11: p. 1280746.

19. Hussain, I., et al., *Routine immunization coverage and immunization card retention in Pakistan: Results from a cross-sectional national survey.* The Pediatric Infectious Disease Journal, 2023. 42(3): p. 260-270.
20. Mvundura, M., et al., *Vaccine wastage in Ghana, Mozambique, and Pakistan: An assessment of wastage rates for four vaccines and the context, causes, drivers, and knowledge, attitudes and practices for vaccine wastage.* Vaccine, 2023. 41(28): p. 4158-4169.
21. Jankowski, P., N. Andrienko, and G. Andrienko, *Map-centred exploratory approach to multiple criteria spatial decision making.* International Journal of Geographical Information Science, 2001. 15(2): p. 101-127.
22. Samarasundera, E., et al., *Methods and tools for geographical mapping and analysis in primary health care.* Primary health care research & development, 2012. 13(1): p. 10-21.
23. Fatima, M., et al., *Geospatial analysis of COVID-19: A scoping review.* International Journal of Environmental Research and Public Health, 2021. 18(5): p. 2336.
24. Hanafi-Bojd, A.A., et al., *Spatial analysis and mapping of malaria risk in an endemic area, south of Iran: a GIS based decision making for planning of control.* Acta tropica, 2012. 122(1): p. 132-137.
25. Raymundo, C.E., et al., *Spatial analysis of COVID-19 incidence and the sociodemographic context in Brazil.* Plos one, 2021. 16(3): p. e0247794.
26. Sun, F., et al., *A spatial analysis of the COVID-19 period prevalence in US counties through June 28, 2020: where geography matters?* Annals of epidemiology, 2020. 52: p. 54-59. e1.
27. Peng, C., P. Goswami, and G. Bai, *A literature review of current technologies on health data integration for patient-centered health management.* Health informatics journal, 2020. 26(3): p. 1926-1951.
28. Vincent, K., et al., *Improving spatial decision making using interactive maps: An empirical study on interface complexity and decision complexity in the North American hazardous waste trade.* Environment and Planning B: Urban Analytics and City Science, 2019. 46(9): p. 1706-1723.
29. Rohwer, A., et al., *Enhancing public health systematic reviews with diagram visualization*. 2021, American Public Health Association. p. 1029-1034.

Figure 1: Stocks and Consumption of Condoms in Actual Quantities

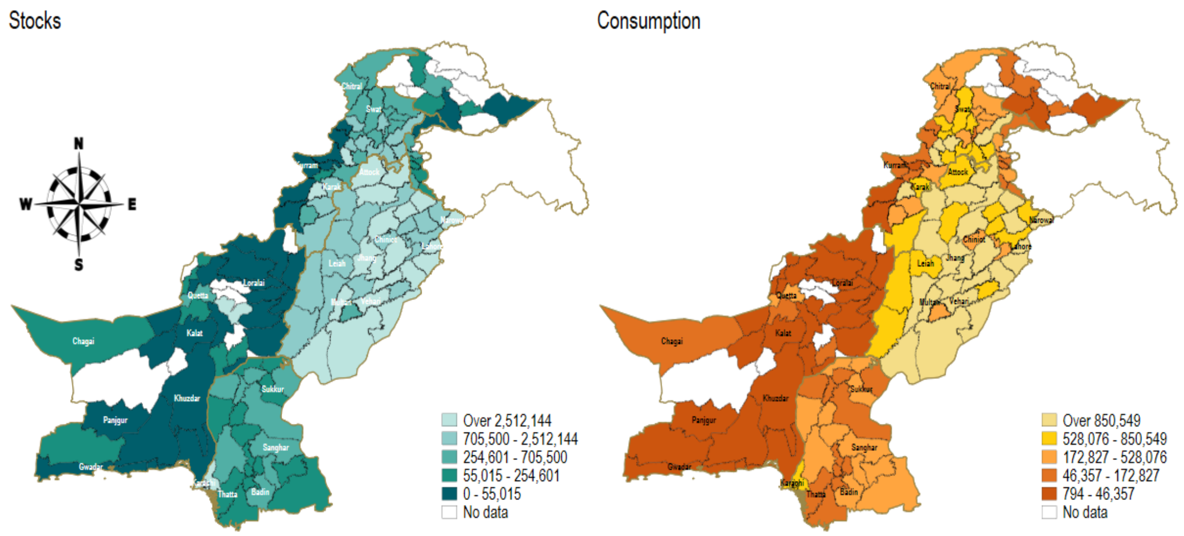

Figure 1: Stocks and Consumption of Condoms Adjusted for Population

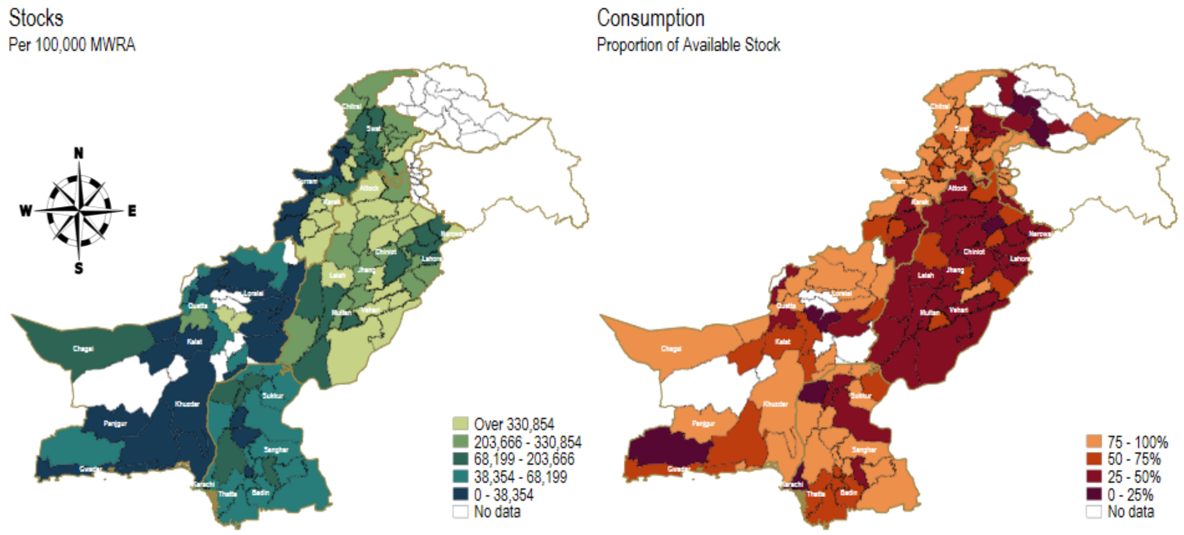

Figure 2: Regional Disparities in Contraceptive Availability and Consumption in Pakistan

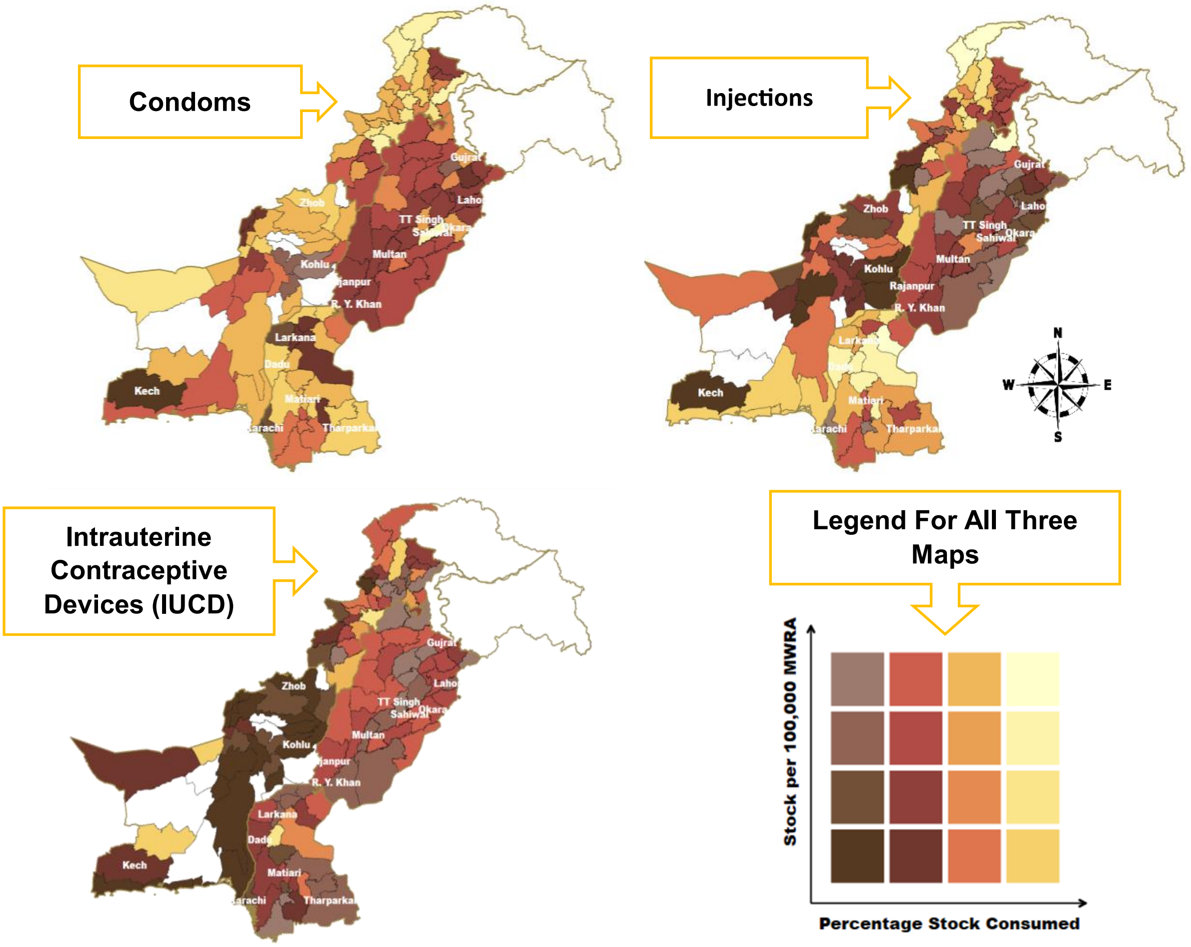

Figure 3: Spatial and Temporal Trends in Outreach per Facility and Penta-3 Dropout Rates in Sindh

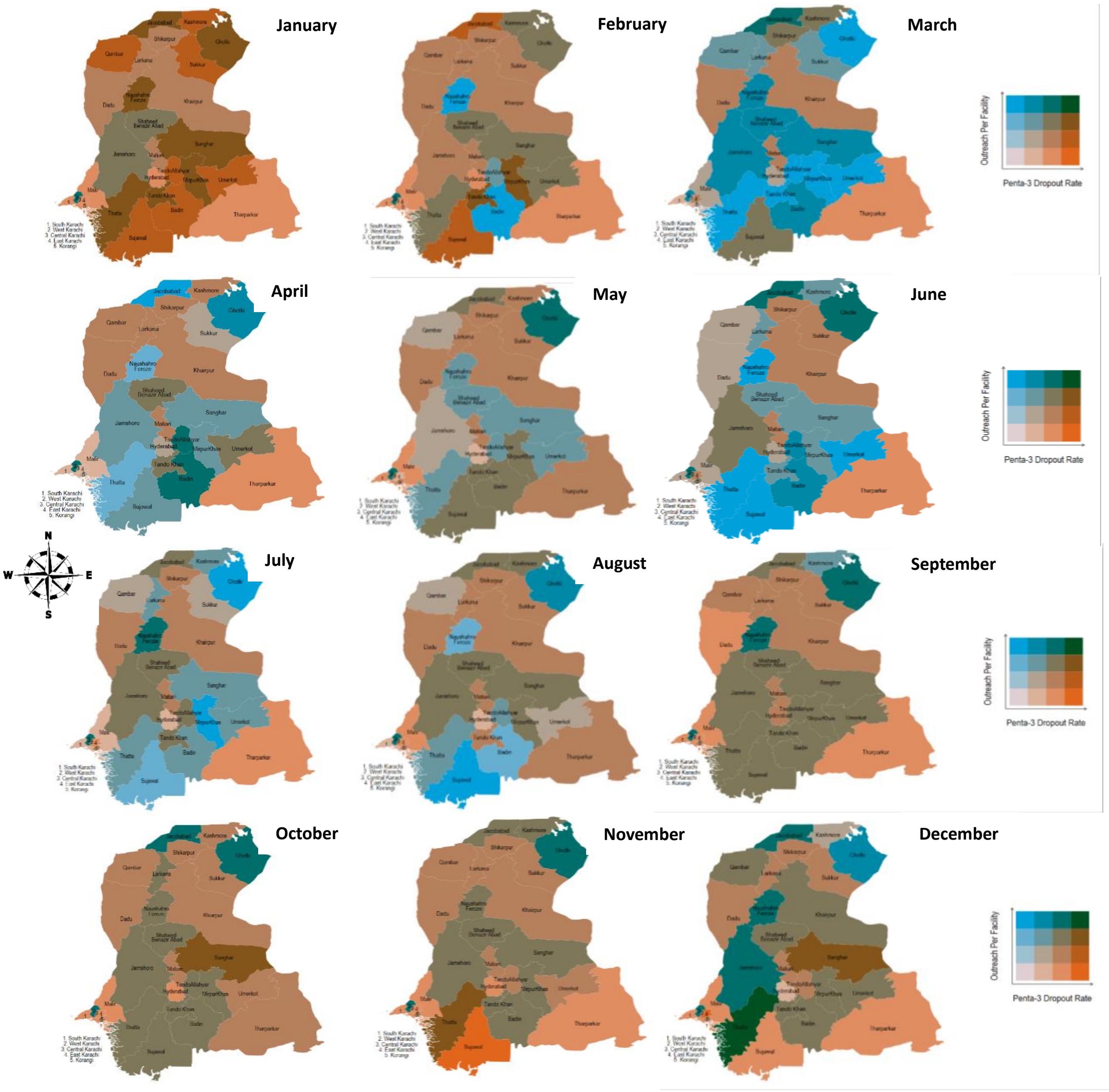

Figure 4: Geospatial Mapping of BCG Vaccine Distribution, Uptake, and Wastage in Sindh

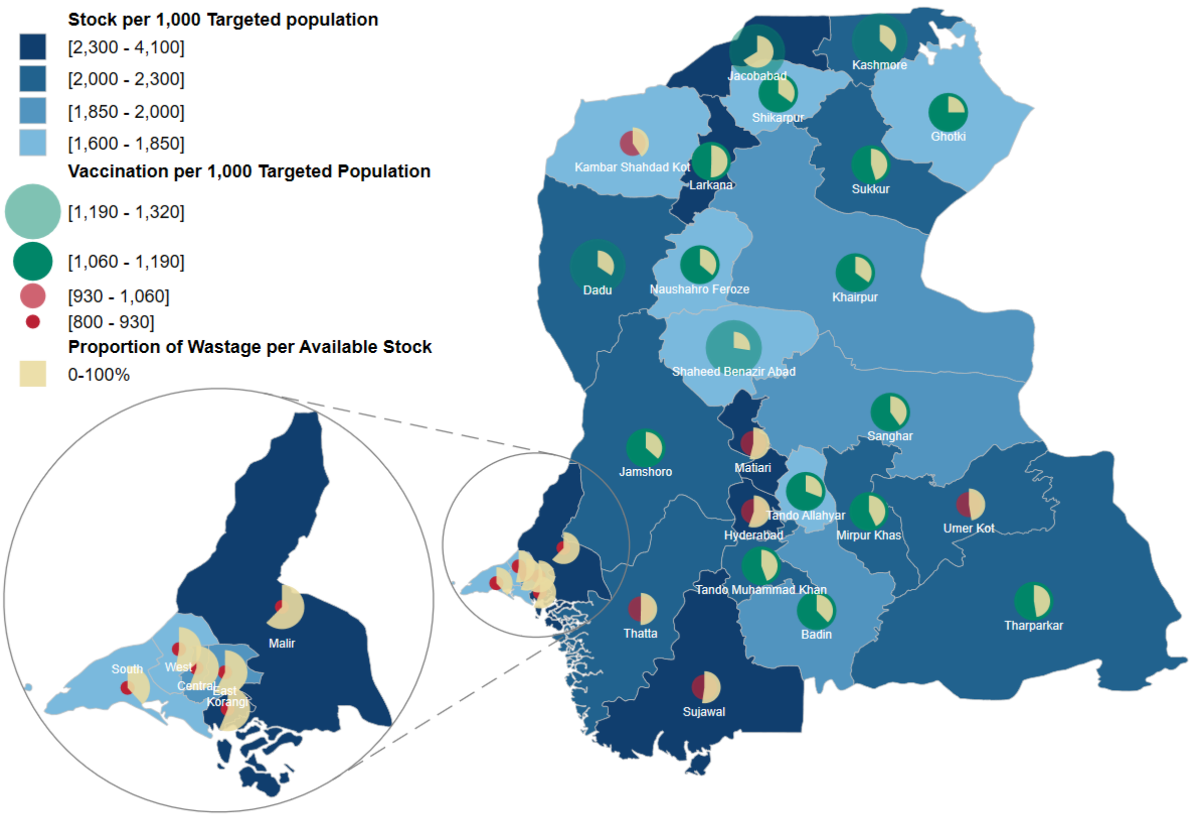

Supplement: Attachment — Submitted filename: PONE-D-25-06311_reviewer.pdf [file pone.0332394.s004.pdf]
