# Supplementary material for: Unlocking insights from complex data: Leveraging heat maps for decision-making in LMIC
Source: PLoS One. 2025 Sep 26;20(9):e0332394. doi: 10.1371/journal.pone.0332394 (PMC12468749; doi:10.1371/journal.pone.0332394)
Supplement: Attachment — Submitted filename: PONE-D-25-06311_ReviewerComments.docx [file pone.0332394.s003.docx]

**Unlocking Insights from Complex Data: Leveraging Heat Maps for Decision-Making in LMICs**

Muhammad Ibrahim^1^, Olan Naz^1^, Amal Fatima Mohiuddin^1^, Habib Ur Rehman^1^, Adnan Ahmad Khan^1,2*^

^1^ Research and Development Solutions (RADS), Islamabad, Pakistan

^2^ Ministry of National Health Services, Regulations and Coordination (MoNHSRC), Islamabad, Pakistan

*Corresponding Author

Adnan Ahmad Khan

Research and Development Solutions

Roshan Aaj Center, Asad Market, I-9/4

Islamabad, Pakistan 44000

Email: [adnan@resdev.org](mailto:adnan@resdev.org)

**Introduction**

Access to effective services is a major to family planning in Pakistan and accounts for over 70% of non-use by those wishing to use FP [1]. A key part of access to effective contraception is the availability of contraceptives at facilities where couples visit. Similarly, inadequate supplies have been a significant constraint in ensuring high vaccine coverage for preventable childhood illnesses. Inability to ensure services and supplies in a timely manner leads to missed opportunities in family planning or other preventive or promotive services such as routine vaccination and exacerbate health outcomes and social disparities. While policy makers seek to address these gaps, it is imperative that their choices are guided by evidence, such how services and supplies are managed and taken up and where are the gaps. Effective utilization of such data can help identify where services and supplies are needed the most, thereby improving health and well-being.

While considerable data are available from healthcare systems, surveys, and various channels, their potential for informed policymaking remains largely untapped [2], due to limited capacity to harness, manage and interpret data, time and consistently [3]. Often, this interpretation would include triangulation of disparate data sources, analysis, and depiction into forms, such as visualization to make data more accessible for decision makers who may not be data experts [4-7]. Additionally, information such as population and program data change rapidly, and interpretations must keep pace with these changes to stay relevant. This underscores the need to utilize adaptable visualization methods that can be replicated routine and frequently to facilitate real-time interpretations [6, 8, 9].

Basic bar graphs and pie charts are often the mainstay of presentations but convey limited information. By contrast, spatial heat maps offer greater flexibility and enhanced readability, effectively illustrating regional variations, highlighting hotspots or time trends [10]. One common advantage of spatial heat maps is that the surface area of country, province or district is always presented through the coordinates present. In addition to depicting simplified indicators, such as stock levels, total consumption, or facility counts, these maps can also simplify more complex visualizations such as the intersection of multi-indicators.

Decision-making is a cognitive process where a person evaluates factors to solve a problem [11], depending on the complexity of the decision (such as the number of criteria and outcomes involved) [12]. While simpler decisions work with simple heuristics, complex ones require solutions that include ranking the quality and utility of data based on predetermined criteria [13]. Limited guidelines illustrate how to design indicators to support complex reasoning across different contexts [14]. Visualizations utilize the brain's sensory capacity to depict complex relationships between data points [15], such as simplifying the relationships of indicators not just across spatial dimensions, but also relate them over time and with other variables [16, 17].

We present one such application in the form of matching supply and demand (two or three factors combined) for contraceptives and vaccines across all districts of Pakistan through spatial heat maps, using data available from the national contraceptive and vaccine logistics management information system (cLMIS and vLMIS respectively). In fact, this technique can be applied to other essential medicines, availability of human resources for health, optimizing various services across or within districts and more. If data are available, the technique can help disaggregate such depictions at more granular levels such as union councils (the smallest administrative unit in Pakistan) or individual facilities.

Data and Methods

Data from the cLMIS and vLMIS portals provided by The Chemonics, through their work with the USAID Global Health Supply Chain Program-Procurement and Supply Management project in Pakistan. Stocks and consumption data for contraceptives were extracted, while vaccine data included indicators such as live birth targets (from now on referred as targeted population), stocks, vaccinations, and wastage. The supply chain for both contraceptives and vaccines is tracked by The Chemonics team from the central warehouse to provincial warehouses, district warehouses, and finally to individual facilities. The focus was on the final stage of supply where stocks are utilized by consumers. Data were aggregated at the district level to create spatial heat maps for broad discussion, which can be replicated at a granular level by district leadership. Monthly data for the calendar year 2022 were aggregated to provide annual estimates for both contraceptives and vaccines, except for one visual used to capture monthly disparities. All spatial heat maps are made using STATA 17.0.

Contraceptives data, comprising only two main variables—stocks and consumption—required careful assessment. Simply displaying these variables on a spatial heat map is insufficient, as it fails to provide meaningful insights. Districts with higher populations naturally require more stock, and consumption alone does not indicate whether the available stocks are being appropriately utilized. For instance, if a district has higher stocks relative to others but a smaller population, and the consumption is below 20%, the information can be misleading. A decision-maker might incorrectly conclude that Married Women of Reproductive Age (MWRA) are not using enough contraceptives, whereas the issue could be overstocking. Therefore, it is essential to standardize these variables for a clearer understanding of the data and look at both indicators together.

To standardize the stocks, the available stock per district was divided by the number of MWRA in a district and multiplied by 100,000 for better understanding of estimates. MWRA numbers were obtained from the Population Census 2017, resulting in the variable “Stocks available per 100,000 MWRA per district” formula shown in Table 1. After 2017, several new districts were added to the cLMIS data which were present in the population census data. Therefore, in the cLMIS data, we aggregated the values for the following districts: Sibi and Lehri, Chitral Lower and Chitral Upper, Kohistan Upper and Kohistan Lower, Duki and Loralai, Killa Abdullah and Chaman, and Shaheed Sikandar Abad and Kalat. Furthermore, cLMIS categorized Karachi as a single entity along with the seven districts within Karachi city, as a result, we aggregated all the data of Karachi city. These values were then categorized into five quantiles. Consumption was standardized by calculating the percentage of available stock consumed per district. This was done by dividing the consumption by the available stock, resulting in four categories: up to 25% of stock consumed (very poor consumption pattern), 25-50% consumed (relatively poor), 50-75% consumed (relatively better), and above 75% consumed. Separately, these spatial heat maps provide limited information, but when analyzed together, they offer more comprehensive insights for targeted policy development. These results are shown in Figure 1 using data for condom contraceptives.

Initially, two separate spatial maps were created to demonstrate how both indicators can be read, although they may be confusing to interpret together. To provide clearer insights, a bi-variate spatial heat map was developed. On the y-axis, stocks per 100,000 MWRA are shown, now divided into four quartiles, with lighter shades indicating higher availability. On the x-axis, color variations represent changes in stock consumption as previously discussed (up to 25%, 25-50%, and so on). This bi-variate structure allows for the examination of sixteen distinct data points, as illustrated in results section (Figure 3). The bi-variate spatial heat maps for condoms, injections, and intrauterine devices (IUDs) are analyzed both separately and all three together. Condoms, being a one-time use contraceptive, show different stock and consumption patterns compared to injections, which provide contraception for one to three months, and IUDs, which last five to eight years. Comparing these three bi-variate spatial maps reveals varying preferences and usage patterns among MWRA (Figure 3).

With the utilization of bi-variate spatial maps established for interconnected indicators, their application is extended to immunization indicators. The focus is on Sindh province, as the Chemonics team validated the data on their portal for this region for vaccinations. There is a notable link between outreach vaccination services and vaccination dropout rates in Pakistan [18, 19]. The Pentavalent vaccine third dose (Penta3) is crucial in protecting children against diphtheria, tetanus, pertussis (whooping cough), hepatitis B, and Haemophilus influenzae type b (Hib). Penta3 vaccination outreach outcomes are analyzed with dropout rates. Calculations of indicators are shown in Table 1. A dropout rate refers to the percentage of children who received the first dose of the vaccine but did not complete the third dose. Monthly graphs were created to display Penta3 doses administered through outreach against Penta3 dropout rates. Demonstrating monthly bi-variate maps reveal significant spatial and temporal variations pertinent to vaccination coverage.

Lastly, since vaccines differ from contraceptives, tri-variate spatial maps were developed to assess vaccine data, incorporating stock availability, consumption, and wastage. Data was taken of Bacillus Calmette-Guerin (BCG) vaccine for 2022 across Sindh. Vaccine wastage is a crucial indicator that complements consumption patterns, [20] referred to as vaccinations. A new visualization strategy was employed to avoid confusion from using multiple colors. This approach integrates information from three variables in an easy-to-understand form. The base color on the spatial map represents stock availability per targeted population. Circles of varying size and color (red indicating low, green indicating adequate) depict vaccination rates per targeted population in each district. Additionally, pie charts overlaid on the circles represent wastage as a percentage of the available stock per district.

The cut-off points for the legend were determined through the quartile’s method, except for the consumed proportion of stocks available where we used percentages as the cutoff point and the wastage rate is a continuous graph starting from 0 to 100%. The cutoff points are shown in the table below (Table 1).

**Table 1: Indicators for Spatial Heat Maps per District**

| Sr. No. | Indicator  (1) | Formula  (2) | Cut-off points of Data in Spatial Maps  (3) | Commodity/Vaccine  (Illustrated in Figure)  (4) |
| --- | --- | --- | --- | --- |
| 1 | Stocks per 100,000 MWRA | $\left( \frac{Total Stocks}{MWRA} \right)\times100,000$ | 0-43,000,  43,000-122,000,  122,000-296,000,  >296,000 | Condoms  (3) |
|  |  |  | 0-5,000,  5,000-13,500,  13,500-21,000,  >21,000 | Injections  (3) |
|  |  |  | 0-1,200,  1,200-3,000,  3,000-6,000,  >6,000 | IUDs  (3) |
| 2 | Consumption Proportion of Stocks Available | $\left( \frac{Total Consumption}{Total Stocks} \right)\times100$ | 0-25%,  25-50%,  50-75%,  75-100% | Condoms, Injections & IUDs  (2 & 3) |
| 3 | Pentavalent-3 outreach per facility | $\left( \frac{Vaccinations through outreach}{No. of Facilities} \right)$ | 0-25,  25-50,  50-75,  >75 | Pentavalent 3  (4) |
| 4 | Pentavalent-3 Dropout Rates | $\left( \frac{Penta1-Penta3}{Penta1} \right)\times100$ | <-50%,  -50-0%,  0-50%,  >50% | Pentavalent 3  (4) |
| 5 | Stocks per 1,000 targeted population | $\left( \frac{Total Stocks}{Live Birth Targets} \right) \times1,000$ | 1,600-1,850,  1,850-2,000,  2,000-2,300,  >2,300 | BCG  (5) |
| 6 | Vaccinations per 1,000 targeted population | $\left( \frac{Total Vaccinations}{Live Birth Targets} \right) \times1,000$ | 800-930,  930-1,060,  1,060-1,190,  >1,190 | BCG  (5) |
| 7 | Proportion of Wastage per Available Stock | $\left( \frac{Total Wastage}{Total Stocks Available} \right) \times100$ | 0-100% | BCG  (5) |

*Note: Above table doesn’t show the cut off points for the univariate spatial maps created for contraceptives, as the values are visible on the legend.*

**Results**

Figure 1Regional Disparities in Contraceptive Availability and Consumption in Pakistan

Distribution and consumption of condoms are used to illustrate the considerable regional variation across Pakistan. Figure 1 describes these in terms of absolute numbers. Central/ eastern regions, particularly Punjab, have the highest stock levels, sometimes exceeding 2.5 million condoms in some districts, while south (parts of Sindh), west (Balochistan) and the northwest (Khyber Pakhtunkhwa – KP) have lower stock levels, ranging from 55-705 thousand condoms per district. Central and northeastern areas display moderate to high stocks, ranging from 0.7 to over 2.5 million. Consumption patterns are similar. The same areas that have the lowest stocks also have the lowest consumption, while those with the highest stocks have the most consumption.

**Figure 1: Stocks and Consumption of Condoms in Actual Quantities**

However, simple depictions of quantity does not account for the need for such commodities. For this, we adjusted the quantities for the population and depicted quantities per 100,000 MWRA (Figure 2). The resulting picture (Figure 2) remains similar for stocks in that, areas that had the least stocks overall also have the lowest stocks when adjected for population. However, the consumption patterns change dramatically. Areas in southwest (Balochistan), south (parts of Sindh) and northwest (parts of KP) that had the least stocks also have the most of their stocks consumed, resulting in stockouts. In the east (most of Punjab), where the more stock was supplied, relatively lesser proportion of the stock was consumed, suggesting oversupply of condoms relative to the population-based need.

**Figure 2: Stocks and Consumption of Condoms Adjusted for Population**

Bi-variate Spatial Maps and importance of its interpretation

This increasing level of complexity from matching supplies and consumption can be depicted in a single figure to give decision makers one composite figure to understand the situation. Figure 3 shows the layering of stocks and consumption together to show the interaction between them. The Y axis shows stocks per 100,000 MWRA, and the X axis shows percent consumption. The figure for condoms confirms that districts in the south, southwest and northeast that have the least supplies also have the least consumption. While in central and eastern districts there are higher stocks and relatively low consumption.

The pattern for contraceptive injections is very similar to condoms. However, the situation is very different for IUCD. There is the least stocking and consumption in Balochistan (west). Sindh (south) sees moderate to high stocks but low consumption while Punjab (central and east) has the highest supplies and low to moderate consumption.

**Figure 3: Regional Disparities in Contraceptive Availability and Consumption in Pakistan**

Spatial and Temporal Insights using Bi-variate Maps

Another layer of information can be incorporated to inform about the effect of time. Figure 4 shows monthly changes in the rate of dropouts for the Penta-3 vaccine as a factor of community outreach across various districts of the Sindh province. There are relatively high dropout rates in many southern and central districts in the first quarter, with some improvements by March. Tharparkar, Sujawal, Dadu and Khairpur consistently show high dropout rates throughout the quarter. Sukkur and Ghotki (in the north) show gradual improvements with relatively lower dropout rates and increasing outreach by the end of the quarter, particularly in March. These patterns can inform about seasonal variations or changes in the level of implementation of interventions over time.

**Figure 4: Spatial and Temporal Trends in Outreach per Facility and Penta-3 Dropout Rates in Sindh**

**Deeper Insights using Three Indicators in Tri-variate Spatial Map**

In addition to the temporal changes the tri-variate spatial map also allows adding a third dimension. For e.g., Sindh province data is displayed for vaccine stock and consumption (both per 1,000 targeted population) vs. the wastage shown as proportion of available stock across districts (Figure 5).

Figure 5 shows that southern (Sujawal, East Karachi, Korangi, Malir, Hyderabad and Matiari) and central (Larkana and Jacobabad) districts have the highest stock levels per 1,000 targeted population, while some northern districts (Kambar Shahdad Kot, Naushahro Feroze) have lower stock levels. Overall, adjusted stock levels show that there are ample stocks available per district. However, vaccination and wastage rates differ across districts.

High vaccination rates are observed in northern and central districts of Sindh such as Jacobabad, Dadu, Kashmore, Shaheed Benazir Abad etc., whereas low vaccination rates in the south (Sujawal, Thatta, and all districts of Karachi), highlighting potential issues in either vaccine distribution or uptake. Significant vaccine wastage is observed, above the 50% threshold, in southern districts such as Sujawal, Hyderabad, and parts of Karachi, indicate inefficiencies in vaccine management. Lower vaccine wastage was observed in central and northern districts (Shaheed Benazir Abad, Naushahro Feroze, Shikarpur, and Ghotki). However, this must be interpreted along with the prior observation of low stocks, suggesting that these lower stocks are being consumed.

**Figure 5: Geospatial Mapping of BCG Vaccine Distribution, Uptake, and Wastage in Sindh**

*Notes: Zoomed part (left part of the Figure, below Legend) are the districts of Karachi City: South Karachi, West Karachi, Central Karachi, East Karachi, Korangi and Malir.*

**Discussion**

Improving data systems and technology, which allows for the collection of increasing amounts and greater granularity of data about health programs, also requires better interpretation techniques. In addition, as more data become available and fewer professionals are able to interpret these data, particularly in LMICs, better techniques can help make data and its interpretation accessible for decision-makers. We present four such techniques.

Simple heat maps represent an improvement over basic tables, particularly when viewing data from large numbers of localities or categories, such as over 150 districts in Pakistan. The next level improvement is to adjust the measure to make it comparable and meaningful, such as the stock amounts adjusted for the population in need in this case. A further layer of complexity was to depict both consumption and supplies in the same figure, using bivariate graphs. Alternatively, changes in indicators over time can be viewed in side-by-side figures that track temporal trends. Finally, interaction of variables such as relationship between the level of outreach and immunization coverage can be explored using tri-variate figures.

Exploratory data techniques effectively support multicriteria spatial decision-making by enhancing the interaction between maps and data graphs/points [21]. The integration of spatial mapping and analysis techniques has enabled the use of an expanding array of routinely available geographic data sources for public health care, research and development [22]. Spatial methods in epidemiology clarify the distribution of population health and its local causes, guiding targeted interventions to prevent disease and promote health [23]. Additionally, recent studies show that spatial heat maps help identify the prevalence and spread of infectious diseases, such as malaria and COVID-19 [24-26]. Our research's spatial heat maps reveal significant regional disparities in the availability and consumption of essential resources across Pakistan, particularly in contraceptives and immunization supplies. This highlights district-level supply and demand issues through specific indicators and underscores the need for resource redistribution across districts with higher stock levels and lower demand.

The analysis of these spatial disparities also facilitates targeted interventions, allowing decision-makers to assess neighboring districts and redistribute resources where needed. For instance, if one district has surplus stocks, these can be redirected to neighboring districts facing shortages. This integration of data helps decision-makers understand the underlying problems better and reduces cognitive complexity by focusing on key factors, thus streamlining the decision-making process. Furthermore, our assessment of temporal-spatial heat maps allows for analyzing patterns over time. For example, Sujawal district (Figure 4) serves as a key example of trend analysis; as outreach services decline, the dropout rate for Penta3 vaccinations increases at specific months. The data underscores the importance of continuous monitoring and adaptive strategies to improve immunization outcomes, particularly in regions with high dropout rates.

Analyzing spatial and temporal trends of the indicator enables us to track its sources within or across districts, while risk maps based on the latest data enhance intervention measures [24]. In Figure 5, a major supply issue is the wastage rate, which we incorporated using tri-variate spatial heat maps. Policymakers should address distribution inefficiencies in districts with high wastage and low vaccination rates, improve storage facilities in areas with high wastage, and enhance community outreach and educational campaigns in regions with low vaccination rates to boost uptake. Frequent use of these visualizations will help analyze large datasets, enabling stakeholders to identify and address potential shortfalls easily.

These visualization techniques provide powerful tools for policymakers, featuring user-friendly interfaces that require minimal technical expertise to generate specific visualizations. By offering detailed, district-level insights into stock levels, consumption, and wastage, these visualizations facilitate data-driven decision-making, ultimately improving program efficiency and resource allocation to meet regional needs. Their scalability ensures a consistent framework for ongoing analysis and monitoring across different districts and over time. They enhance data clarity and accessibility, contributing to the continuous improvement of public health programs. Bivariate or trivariate spatial heat maps help identify areas that require stock adjustments, outreach service enhancements, and wastage reduction, providing valuable tools for policy formulation and implementation. This approach not only improves the efficiency of contraceptive and immunization programs but also ensures that resources are allocated appropriately to meet the needs of various regions.

Once decision-makers identify the concept and analysis syntax, they can embed analysis leading to such spatial heat maps directly into the database or data dashboards using short programs called Application Programming Interfaces (APIs) [27]. Furthermore, interactive maps can significantly enhance spatial decision-making [28] upon clicking the district decision can get statistical of that district. This enables decision-makers to generate them monthly or more frequently with a single click, eliminating the need for complex data analysis. Our district example can be replicated for the entire country, a single province, or even an individual facility. We can also extend the process to other commodities, such as medicines, supplies, or even human resources for health. If we manage these commodities on the same dataset, the process simplifies by allowing users to click on filters from a drop-down menu [29].

Such tools facilitate analysis at both the macro and micro levels, helping decision-makers identify overstocked or understocked locations, understand demand patterns, and identify resources for redistribution between neighboring districts or facilities, leading to informed decisions. Our findings contribute to visualizations that allow users to query large datasets with minimal human expertise, enabling better resource allocation at health facilities and ultimately improving health outcomes.

**Conclusion**

Our analysis of contraceptive and immunization data using bi-variate and tri-variate spatial heat maps reveals significant regional disparities in Pakistan. Urban centers, particularly in developed areas like Punjab and Sindh, show better stock availability and higher consumption rates than less developed regions such as Balochistan and southern Khyber Pakhtunkhwa. In immunization, our temporal and spatial analysis of Penta3 indicator highlights seasonal trends and persistent outreach challenges in many parts of Sindh province. This necessitates focused and resource-intensive strategies to improve vaccination coverage. Addressing vaccine wastage through improved distribution and storage, along with enhanced community outreach, is crucial.

Integrating data point and spatial maps enhances decision-making processes. These visualizations offer detailed insights into resource distribution, facilitating data-driven decisions that optimize program efficiency. Spatial maps simplify the generation of actionable insights, empowering policymakers to make informed decisions quickly. Ultimately, spatial maps serve as essential tools for guiding health interventions, ensuring effective resource allocation to meet diverse regional needs. This research highlights the critical role of spatial analysis in improving public health outcomes through targeted policy actions.

**Acknowledgment**:

We would like to extend our gratitude to CHEMONICS Pakistan for providing the valuable data used in this study. Their contribution has been instrumental in enabling our research and enhancing our understanding of the supply and consumption dynamics of contraceptives in Pakistan.

**References**

1.	(NIPS), N.I.o.P.S., *PAKISTAN DEMOGRAPHIC AND HEALTH SURVEY 2017-18*. 2018.

2.	Roberts, J., et al., *From Ill-Defined Problems to Informed Decisions.*

3.	Aung, T., et al., *Optimizing data visualization for reproductive, maternal, newborn, child health, and nutrition (RMNCH&N) policymaking: data visualization preferences and interpretation capacity among decision-makers in Tanzania.* Global Health Research and Policy, 2019. 4: p. 1-14.

4.	Aung, T., D. Niyeha, and R. Heidkamp, *Leveraging data visualization to improve the use of data for global health decision-making.* Journal of global health, 2019. 9(2).

5.	Zakkar, M. and K. Sedig, *Interactive visualization of public health indicators to support policymaking: An exploratory study.* Online journal of public health informatics, 2017. 9(2).

6.	Raghupathi, W. and V. Raghupathi, *An empirical study of chronic diseases in the United States: a visual analytics approach to public health.* International journal of environmental research and public health, 2018. 15(3): p. 431.

7.	Sadiku, M., et al., *Data visualization.* International Journal of Engineering Research And Advanced Technology (IJERAT), 2016. 2(12): p. 11-16.

8.	Lavrač, N., et al., *Data mining and visualization for decision support and modeling of public health-care resources.* Journal of biomedical informatics, 2007. 40(4): p. 438-447.

9.	Preim, B. and K. Lawonn. *A survey of visual analytics for public health*. in *Computer Graphics Forum*. 2020. Wiley Online Library.

10.	Słomska-Przech, K., T. Panecki, and W. Pokojski, *Heat maps: Perfect maps for quick reading? comparing usability of heat maps with different levels of generalization.* ISPRS International Journal of Geo-Information, 2021. 10(8): p. 562.

11.	Wang, Y. and G. Ruhe, *The cognitive process of decision making.* International Journal of Cognitive Informatics and Natural Intelligence (IJCINI), 2007. 1(2): p. 73-85.

12.	Jelokhani-Niaraki, M. and J. Malczewski, *Decision complexity and consensus in Web-based spatial decision making: A case study of site selection problem using GIS and multicriteria analysis.* Cities, 2015. 45: p. 60-70.

13.	Einhorn, H.J. and R.M. Hogarth, *Rationality and the sanctity of competence.* Behavioral and Brain Sciences, 1981. 4(3): p. 334-335.

14.	MacEachren, A.M. *Distributed cognition: A conceptual framework for understanding map-based reasoning*. in *Proceeding of the 27th International Cartographic Conference (August 2015), International Cartographic Association.* [*http://icaci*](http://icaci)*. org/files/documents/ICC proceedings/ICC2015/papers/2/940. html*. 2015.

15.	Roth, R.E. and A.M. MacEachren, *Geovisual analytics and the science of interaction: an empirical interaction study.* Cartography and Geographic Information Science, 2016. 43(1): p. 30-54.

16.	Jankowski, P. and L. Richard, *Integration of GIS-based suitability analysis and multicriteria evaluation in a spatial decision support system for route selection.* Environment and Planning B: Planning and Design, 1994. 21(3): p. 323-340.

17.	Leung, Y., *Intelligent spatial decision support systems*. 2012: Springer Science & Business Media.

18.	Shiferie, F., et al., *Vaccination dropout and wealth related inequality among children aged 12–35 months in remote and underserved settings of Ethiopia: a cross-sectional evaluation survey.* Frontiers in Pediatrics, 2023. 11: p. 1280746.

19.	Hussain, I., et al., *Routine immunization coverage and immunization card retention in Pakistan: Results from a cross-sectional national survey.* The Pediatric Infectious Disease Journal, 2023. 42(3): p. 260-270.

20.	Mvundura, M., et al., *Vaccine wastage in Ghana, Mozambique, and Pakistan: An assessment of wastage rates for four vaccines and the context, causes, drivers, and knowledge, attitudes and practices for vaccine wastage.* Vaccine, 2023. 41(28): p. 4158-4169.

21.	Jankowski, P., N. Andrienko, and G. Andrienko, *Map-centred exploratory approach to multiple criteria spatial decision making.* International Journal of Geographical Information Science, 2001. 15(2): p. 101-127.

22.	Samarasundera, E., et al., *Methods and tools for geographical mapping and analysis in primary health care.* Primary health care research & development, 2012. 13(1): p. 10-21.

23.	Fatima, M., et al., *Geospatial analysis of COVID-19: A scoping review.* International Journal of Environmental Research and Public Health, 2021. 18(5): p. 2336.

24.	Hanafi-Bojd, A.A., et al., *Spatial analysis and mapping of malaria risk in an endemic area, south of Iran: a GIS based decision making for planning of control.* Acta tropica, 2012. 122(1): p. 132-137.

25.	Raymundo, C.E., et al., *Spatial analysis of COVID-19 incidence and the sociodemographic context in Brazil.* Plos one, 2021. 16(3): p. e0247794.

26.	Sun, F., et al., *A spatial analysis of the COVID-19 period prevalence in US counties through June 28, 2020: where geography matters?* Annals of epidemiology, 2020. 52: p. 54-59. e1.

27.	Peng, C., P. Goswami, and G. Bai, *A literature review of current technologies on health data integration for patient-centered health management.* Health informatics journal, 2020. 26(3): p. 1926-1951.

28.	Vincent, K., et al., *Improving spatial decision making using interactive maps: An empirical study on interface complexity and decision complexity in the North American hazardous waste trade.* Environment and Planning B: Urban Analytics and City Science, 2019. 46(9): p. 1706-1723.

29.	Rohwer, A., et al., *Enhancing public health systematic reviews with diagram visualization*. 2021, American Public Health Association. p. 1029-1034.
