# Supplementary material for: Unlocking insights from complex data: Leveraging heat maps for decision-making in LMIC
Source: PLoS One. 2025 Sep 26;20(9):e0332394. doi: 10.1371/journal.pone.0332394 (PMC12468749; doi:10.1371/journal.pone.0332394)
Supplement: Attachment — Submitted filename: Response to Reviewers.docx [file pone.0332394.s006.docx]

We thank all the reviewers for the detailed review of the manuscript. Highlighting the grammatical errors and suggesting changes for better research document. We appreciate the remarks regarding the importance of this research.

**Comment for all reviewers**

We had to change the shape files to meet the requirements of PLOS One journal. Due to this change, we had to make some modifications in estimates for few districts which has mentioned on page 8 (Data and Methodology section, subsection Data Standardization, paragraph 2). Two districts each from Punjab and Sindh were a part of other districts so we merged the estimates for the specific districts. Due to this, contraceptives cut off points were changed, however, for immunization we kept the same cutoff points as those were custom cutoff points based on the data, there was little variation in data points for Sindh, hence, we kept the same cutoffs. Secondly, we have removed any results from the specific districts in the results and discussion sections, revised the results according to the districts available in Shape files.

**Reviewer 1 comments:**

| **Reviewer Comment** | **Reply** |
| --- | --- |
| Congratulations on preparing a well-structured and logically coherent manuscript. The document is well written; however, some revisions are necessary to enhance its clarity and completeness. The reviewer's suggestions and comments are noted in the attached document for your consideration.  It appears that the final manuscript was not thoroughly reviewed before submission, and certain sections would benefit from further elaboration and/or clarification. Additionally, the references do not adhere to a standard format and should be revised accordingly.  I am recommending minor revisions, after which the manuscript can be resubmitted for publication. Wishing the authors the best of luck in the revision process. | Thank you for your appreciation and for identifying the grammatical errors in the manuscript. We have carefully revised the text and made the necessary corrections. Where additional context was required, we have elaborated accordingly to enhance clarity and ensure the content is more comprehensible. |
| **Main comments from the document** |  |
| Add references | We have added relevant references in the first paragraph  Suggested reference was added in the second paragraph  Stata reference is added |
| Elaboration required in the third paragraph for following sentence: “One common advantage of spatial heat maps is that the surface area of country, province or district is always presented through the coordinates present.” | Elaborated sentence: “One key advantage of spatial heat maps is that they consistently represent the surface area of a country, province, or district using geographic coordinates, ensuring accurate spatial context and allowing for clear comparisons across regions.” |
| Consistency with the directions | We have added additional information to clarify where we mean south of Pakistan and then where we talk about direction within a province.  Thank you for highlighting additional districts which we missed out in the manuscript |
| Add specific months for temporal graph | It was the winter season that had major dropout rates for Penta3. We have added that in the discussion section. |
| Consumption patterns for contraceptives in Punjab | We have edited that in the conclusion section, highlighting the stocks level only. |
| Referencing list needs revision | We have revised the reference list and made the relevant changes |

**Reviewer 2 comments:**

| **Reviewer Comments** | **Reply** |
| --- | --- |
| The manuscript introduces an interesting approach towards analysing contraceptive availability and consumption data along with vaccine availability and administration in Pakistan. This approach per se is not novel, however, provides some level of insight into these issues in the context of Pakistan. The manuscript does not fully justify how and why the performed analysis may be superior to techniques already deployed and in use. | Thank you for your insightful comment. In response, we have revised the objective and aim paragraph in the introduction section to provide clearer context for our approach. Additionally, we have expanded the methods section to include more detailed information, ensuring further clarity on how and why our analysis offers valuable insights. We have also revisited the discussion section to address these points more thoroughly. We believe these revisions will help better highlight the rationale behind our approach and its potential contribution to the field. |
| **Abstract** |  |
| The authors mention the effect of inputs on “rate of utilization of services”. The paper does not mention any rate(s) either for contraceptive use or for immunization. | Thank you for your comment. We agree that framing inputs in terms of the "rate of utilization of services" may cause confusion for readers. Our approach does not directly address rates of utilization but rather focuses on the availability of stocks, adjusted for population size, and the extent to which these stocks are being consumed. Our primary aim is not to present numerical rates but to provide visualizations that can assess whether certain districts have sufficient stock relative to others and whether the available stock is being consumed at adequate levels. This approach helps identify potential demand-side issues related to stock availability.  In response to your feedback, we have revised the text in the abstract to clarify this and now refer to it as the "utilization of contraceptives and vaccines." This should better align the manuscript with the intended focus and provide clearer context for the reader. |
| The objective appears to be “We explore the use of spatial heat maps to analyze the distribution of contraceptives and vaccines in Pakistan and to depict these analyses as visualizations.” However, the data from cLMIS is merely the stock of contraceptive(s), not distribution. How do the authors plan to address this aspect? | Thank you for your comment. You are correct that the data from cLMIS pertains to the stock of contraceptives, not the distribution per se. In our approach, we use "distribution" in the sense of how stock is available across different districts. Once this availability is highlighted, the visualization can help decision-makers understand where stocks are concentrated and identify potential redistribution of stocks opportunities between districts.  However, we recognize that this phrasing might be confusing for readers. In response, we have revised the objective to clarify this point and now refer to it as "stock availability and consumption/vaccination patterns of contraceptives and vaccines." This revision aligns with your suggestion to be more specific and accurately reflects the focus of our analysis. |
| The results section states “we also show the effect of inputs (supplies, outreach) on rate of utilization of services (contraceptive uptake and vaccine coverage). Finally we depict how these visualizations can help track changes in programming over time.” This is merely a claim and not the finding or result of the study. For example, what is the “rate of utilization of services” the authors have measured? What are the findings that help track change over time? | Thank you for your comment. The "rate of utilization of services" in our study is directly associated with the percentage of stocks consumed, which is calculated as the ratio of stocks consumed to stocks available. We acknowledge that this clarification was not sufficiently highlighted in the results section.  Regarding tracking changes over time, the example provided for contraceptives is not explicitly presented in our study. However, we have demonstrated this aspect through the immunization section (Figure 4), where we show how indicators can be assessed over time for improved outcomes. This example illustrates how our approach can be applied to track changes and assess performance over time.  Furthermore, we utilized two domains in this paper—contraceptives and immunization—to show that this approach is versatile and can be applied across different sectors, not just limited to contraceptive or immunization data. We hope this clears up the confusion and provides a more accurate reflection of our findings. |
| The authors conclude that they the study “identifies critical gaps in health service supply and demand”, but it is not clear what is the gap? | Each figure in the manuscript highlights a specific issue using underutilized but available data. We have structured the paper to demonstrate how a novel visualization-based approach can offer fresh insights, particularly in contexts where conventional methods may not fully capture programmatic gaps. For contraceptives (Figures 2 and 3), we present the supply side through population-adjusted stock availability, while the proportion of that stock consumed serves as a proxy for demand. When we refer to a "gap," stocks at facility based on conjectured population figures in some distant past and not necessarily updated over time or based on census or other data. We show pattern of utilization and correlation with other districts, changes over time. Allowing policy-makers on present time available data.  This has addressed in the objective paragraph of the introduction in the main body for better clarity. |
| **Specific Comments** |  |
| There is a question of generalizability of this study. The authors have referred to its application to all LMICs in the conclusion. However, does Pakistan reflect all LMICs, more so since vaccination data analysis covers the province of Sindh and that too for BCG and Penta-3 via outreach facilities only? | In the last section of introduction, we have added the focus is on Pakistan for the study, not all LMIC. Hence, Pakistan serves as an example for this study. |
| Please revisit the introduction section for more clarity. There are various language errors, note lines 35, 39, 48, 53, 59, 61, 69 and others. | All the grammatical errors have been addressed in the manuscript. Thank you for highlighting these. |
| Lines 74-78 appear to be more of a discussion point rather than introduction. | Agreed. Lines are removed from Introduction and moved to discussion |
| Objective is not clear in the introduction section. | Objective has been edited and clarified in the introduction section. |
| More information on facilities which report data both for cLMIS and vLMIS will be helpful. Are these facilities all in the public sector or in the private sector as well. For contraceptive data, are the facilities operated by the health departments or the population departments in respective provinces. Is contraceptive availability captured in from the private sector as well. Does only outreach Penta-3 coverage provide sufficient information for this kind on analysis? | Data is collected from all the public and private service providers. In public sector, population welfare department’s data is captured separately, and health department data is collected separately. Same is true for immunization data. We have added a line explaining that data is collected from all the public and private service providers.  This is one illustrated approach, and by no means the only one. This sort of analysis can be done for any vaccine but also allow correlation with non-stock factors such as outreach or any other social or logistical determinant. For example, it can even be used to assess the dropout rates for measles.  Secondly, outreach was selected for Penta3 because there is correlation between higher dropout rates and low outreach activities. In methods section, we have added a reference for this association. |
| For contraceptives the authors have stated that the consumption data does not mean that contraceptives have been utilized (line 97). In this situation how can it be suggested that rates of utilization of services have been measured (see abstract). | Thank you for your thoughtful comment. The statement on line 97 clarifies that consumption data alone does not necessarily reflect appropriate or effective utilization of contraceptives. This is because raw consumption figures, without context, do not account for factors such as population size or stock availability. To address this, our approach adjusts consumption relative to available stock, which itself is adjusted for population. By interpreting these two indicators together—population-adjusted stock availability and the proportion of that stock consumed—we aim to provide a more meaningful understanding of service utilization. Accordingly, we have revised the abstract to better reflect this nuanced approach to interpreting utilization patterns. |
| It is unclear from the analysis if private sector information is part of this data set. Implications will be different if it is than if it is not. Please ensure that it is made clear. | Yes, that is added in the methods section now. |
| Discussion section needs to be revisited. It will benefit from a discussion around the findings, that is how does the spatial analysis report the status of the contraceptive and vaccine availability as well as consumption. What are the regional disparities? A discussion of trends. | We have added a paragraph in discussion section, highlighting some key findings |
| Please also discuss potential limitations in some detail. | Added a section on limitation |
| The authors state that, “policymakers should address distribution inefficiencies in districts with high wastage and low vaccination rates, improve storage facilities in areas with high wastage, and enhance community outreach and educational campaigns in regions with low vaccination rates to boost uptake.” How is this recommendation flow from the spatial analysis conducted in this study? Isn’t this already known, BCG has one of the highest wastage rates among vaccines. | Yes, BCG has 50 percent wastage rate known by administrative staff. Through our analysis we can see that there are districts with higher than 50 percent wastage rate. Data also suggests that there are districts with less than 50 percent wastage rate which means there is a possibility to reduce wastage rates as well if the distribution inefficiencies are catered for.  Hence, our tri-variate spatial map tries to capture this on a visual for better understanding. |
| What policy level decisions can be taken by policy makers using this approach of data analysis? | Government can intervene into the districts where demand of contraceptives is low and redistribution of stocks from districts with higher stocks availability to districts where there is less stock available. Line numbers: 336 – 356.  We are not implying specific changes the government should make through these figures. The approach for this paper is to introduce a way through which policy makers can make some logistical decisions, like red flag a district for not performing well.  We did not deep dive into the problems as this approach only highlights the problem at quicker pace for the policy makers. Rectifying the problem would require deeper dive into each district and solve the demand or supply issue which can be monitored through this approach. The progress of the intervention can be also monitored. |
| The discussion section talks about some dashboard and policy makers clicking to get statistics of a district. Was that the purpose of this study? It is not relevant and confuses the reader. | Thank you for your comment. The reference to dashboards in the discussion section was included to suggest potential future applications of our approach. Specifically, we intended to illustrate how spatial visualizations, like the ones presented in our study, could be integrated into interactive dashboards—allowing policymakers to click on a district to access additional, relevant indicators to support data-driven decision-making. However, we understand that this may have appeared tangential to the core objective of the study. We have revised the text to clarify that this is a proposed implication for future work, not the primary purpose of the current analysis. |
| Discussion needs to be crisp and avoid repetition which is considerable at present. | We agree and discussion has been thoroughly checked for any errors or repetition. |
| The manuscript does not convince the reader how the use of visualizations may improve routine data analysis and interpretation in this context. | We have added additional information in explaining the graphs and its implication for readers in the introduction to see how these visualizations will assist the policy maker |

**Reviewer 3 comments:**

| **Reviewer Comments** | **Reply** |
| --- | --- |
| It is an immensely useful topic to investigate for country with massive population growth and sub-optimal policy development process.  Major areas of improvement for this mansucript are language and grammatical errors, logical flow and failure to acknowledge the practical and methodological limitations. | We have gone through the manuscript and corrected all grammatical errors and fixed the logical flow. Added limitations section for the practical and methodological limitations |
| 'Consumption' is a key variable and intricate details are lacking, how it is calcuated. | Consumption is calculated as the number of commodities consumed from the facility. We have added this definition in the methods section. |
| **Comments from the PDF document** |  |
| Aim is not convincing and specific | We have revised the final paragraph of the introduction to provide greater clarity on the study’s objective and to articulate the aim more specifically. |
| Vaccine stocks and management is barely mentioned in the intro  StudyData collection time period is not stated explicitly. | We intentionally structured the visualizations to enable readers to interpret the graphs and derive insights independently, highlighting the value of visual analytics in informing decision-making. While a more detailed analysis of vaccine stock management could offer additional context, it falls beyond the scope of this study, which is centered on demonstrating the diagnostic potential of spatial visualization tools. As suggested, this point has now been acknowledged in the limitations section. |
| how assessed and recorded, by whom, likelihood of missing information or inconnect values | We have added information about missing information incorrect values as a limitation in the methods section and Limitation section as well. |
| Suggest to dedicate the methods section to 'what' and 'why' presented in discussion section.  So this section better be pulled up removing the rationale given before | We have incorporated the 'what' and 'why' components into the Methods section, as suggested. Upon revisiting the section, we agree that it lacked clarity in its original form. To improve readability and coherence, we have introduced subsections to better reflect the analytical flow followed in the study. |
| This could be potentially true but this cross sectional study cannot establish the direction of the relationship | Thank you for your observation. We fully agree that, as a cross-sectional study, our analysis cannot establish the direction of the relationships observed. We would like to clarify that the inferences drawn are specific to the year analyzed and are not intended to suggest consistent trends over time. Our primary objective is to demonstrate how spatial maps can serve as diagnostic tools to support timely, data-driven interventions by providing policymakers with a clear and comprehensive snapshot on an annual or monthly basis.  Additionally, while the contraceptive analysis was cross-sectional, we included temporal spatial maps in the immunization section to illustrate how such tools can support temporal inferences. Similar approaches could be applied to contraceptive data if monthly trends were to be explored. We deliberately used distinct examples from different health domains—contraceptives and immunization—to highlight the versatility of spatial mapping methods. However, the same analytical approaches can be readily extended to other areas of public health. |
| There is no limitation section and two limitations must be mentioned and discussed:  - Cross sectional nature of the study  - Ecological fallacy  Conclusion must take these into account  Due to the lack of time dimension, this analysis serves as a motivation to initiate exploring the unmet need mitigation planning. | Thank you for your valuable feedback. A dedicated limitations section has now been added to the manuscript. As suggested, we have explicitly addressed the cross-sectional nature of the study and the potential for ecological fallacy. We acknowledge that our visual approach highlights patterns and disparities in the current system but does not allow for causal inferences or individual-level conclusions. This underscores the need for further in-depth research to explore underlying causes and inform programmatic responses.  While we did not include a temporal analysis for contraceptives, we demonstrated this approach through immunization data, illustrating how spatial maps can capture changes over time. This method can be readily extended to contraceptive data, and our narrative uses examples from both health domains to showcase the broader applicability of spatial visualization tools. Ultimately, the analysis aims to serve as a starting point to inform and motivate targeted planning to address unmet needs. |
| Bivariate helps communicating case development but the conclusions better focus only be based on tri-variate results. | Thank you for your comment. Our approach, however, involves the use of bi-variate or tri-variate analysis, depending on the context and the availability of data. In the case of immunization, wastage is an additional variable that needs to be assessed. Unfortunately, no wastage data was available for contraceptives, which is why we opted to use heat maps with only two variables.  Regarding the graphs presented, we believe that the graphs shown in Figure 1 are not particularly useful in our context. In contrast, we consider each graph in Figure 2 to be a valuable monitoring tool. |
| Incomplete reference | Thank you for highlighting that, we have corrected our references list. |
| Figures are the meat of this paper yet are of poor resolution and minimally labeled. Instead of names, numbers can be used to identify districts and a table can accompany to link the number with the district name. | Thank you fir highlighting this, Resolution of the figures were reduced automatically in the word file when we uploaded on PLOS one journal. We adjusted this setting on word file and now you can see high resolution figures, hopefully. If any problem arises, do let us know.  Secondly, we have labelled all the districts in the map now. We have kept the size of labels small, which can be read in high resolution figure. For few districts, we took your advice of numbering them and adding a caption for the reader. We did not add a table because our main narrative remains to use visualization rather than tables for assessment and including table in any way can cause confusion for reader. We hope you understand our point. |
