# Supplementary material for: Unlocking insights from complex data: Leveraging heat maps for decision-making in LMIC
Source: PLoS One. 2025 Sep 26;20(9):e0332394. doi: 10.1371/journal.pone.0332394 (PMC12468749; doi:10.1371/journal.pone.0332394)
Supplement: Attachment — Submitted filename: Response_to_Reviewers.docx [file pone.0332394.s007.docx]

Response to Reviewers

| Reviewer Comment | Author Response |
| --- | --- |
| Reviewer 2: Reporting on 'consumption'. While the authors have tried to address this by adding a definition for consumption, by their own admission "consumption alone does not indicate whether the available stocks are being appropriately utilized" (178-179). This contradicts with their assertion that "the proportion of stocks consumed serves as an indicator of demand for available  commodities. For example, if consumption falls below 25% or 50% of the available stock, it suggests under utilization and potential misalignment between supply and demand" (208-210).  The fundamental point is that cLMIS data does not provide consumption information for contraceptives such as condoms and oral pills. This is only 'issuance' or 'dispensation' data. Once a client is given these contraceptives, only clients can provide the information on whether the method has been utilized or otherwise. This is a serious limitation in this analysis and has not been discussed in the relevant section. | We appreciate this important observation. We thank the reviewer for highlighting this important limitation. In the revised manuscript (Methods and Limitations sections), we have clarified that the variable labeled as “consumption” in the LMIS dataset actually refers to *dispensation*, i.e., the issuance of commodities from facilities to clients. This does not capture actual utilization, which can only be determined from client-side data. Consequently, we now consistently use the term *dispensation* when referring to LMIS-derived measures and explicitly state that these data reflect supply-side transactions, not verified end use. The Limitations section now notes that the absence of utilization data may overestimate true uptake. |
| Reviewer 2: Reporting 'trends'. The data represented from a single year i.e. 2022. The only trends provided are for Penta-3 vaccine and that too only for 12 months in a single year. The paper is presenting this as a trend analysis for contraceptives as well, which is not correct. | Thank you for highlighting this error in our manuscript. We have revised the manuscript, calling it variations over time in most parts of the manuscript, as rightly pointed out. We tried to address the misconception of showing any over time analysis for the contraceptives as well. |
| Reviewer 4: The study applies heat‐map visualizations coherently and the data presented support the stated conclusions about geographic disparities and temporal trends in contraceptive and vaccine logistics. However, the manuscript lacks a more rigorous description of any statistical testing or validation of the quantile cut‐offs and does not discuss uncertainty or sensitivity of the maps. | We have now revised the Methods section to clarify that quartile-based classification was used to account for the skewed distribution of stock-to-population ratios and to enable meaningful cross-district comparisons. We also added results from sensitivity analyses comparing quartile and equal-interval methods, which showed that equal intervals obscured mid- and lower-range differences. Furthermore, we specify that for dispensed stock and Pentavalent-3 dropout rates, fixed percentage thresholds were used due to the continuous nature of these indicators. The relevant revisions are now reflected in the updated manuscript |
| Reviewer 4: While the mapping methods are clearly described, no statistical tests (e.g., spatial autocorrelation measures, significance of differences between regions, or confidence intervals for consumption proportions) are reported. The choice of quartile cut‐offs should be justified or tested for robustness. | We really appreciate the reviewer’s suggestion. In the revised manuscript, we have added formal spatial autocorrelation analyses for key contraceptive commodities (condoms, injections, and IUDs) using Local Moran’s I statistics. These were calculated using queen contiguity spatial weights derived from district boundaries. For each commodity, we examined univariate clustering for stocks and dispensation, as well as bivariate Local Moran’s I to assess spatial relationships between stocks and dispensation, and vice versa.  The results, summarized in Table 2 and shown in the LISA cluster and significance maps in the Appendix, quantify and test the statistical significance of spatial clustering patterns. |
| Reviewer 4: The Data Availability Statement indicates that data “will be available upon request” from Chemonics, which does not satisfy PLOS ONE’s requirement for fully open data. The authors must deposit cleaned, de‐identified datasets and any shapefiles to a public repository (e.g., Dryad, Zenodo) and include accession DOIs. | Chemonics data is available on special request, as Chemonics collects data from government entities and private entities, they do not provide open access to the data. However, we have added information about the shapefiles that is readily available. |
| Reviewer 4: Overall the English is clear and the flow logical. A few grammatical or typographical errors remain (e.g., “adjected” → “adjusted” in Figure 2 caption; inconsistent hyphenation of “bi-variate” vs. “bivariate”). The Introduction and Discussion could be tightened to avoid some repetition. | These typographical errors have been corrected. 'Adjusted' has replaced 'adjected', and 'bivariate' and ‘trivariate’ are now used consistently throughout the manuscript without any hyphenation.  Introduction and Discussion have been tightened to avoid any repetition. |
| Reviewer 4: Remove duplicate paragraphs in Discussion beginning 'Improving data systems…' and 'Exploratory data techniques…' | We have removed the duplicate paragraphs in the Discussion section to improve clarity and flow. Keeping second now as our first paragraph in the discussion section. And removed any other duplication of points |
| Reviewer 4: Although this is secondary data, state explicitly 'Data are aggregate and anonymized; ethics approval was not required' | We have updated the Ethics Statement to clarify that the data are aggregate and anonymized, and that ethics approval was not required.  It was instructed by PLOS one Production Team to give caption to each figure about the CC BY License, where we have used the shapefiles. Hence, we have added that information below each map. |
| Reviewer 4: Limitations section should discuss potential biases due to delayed reporting, data entry errors, and the exclusion of private sector LMIS gaps. Also note that MWRA estimates are based on the 2017 census only. | We have expanded the Limitations section to include potential biases such as delayed reporting, data entry errors, exclusion of private sector LMIS data, and the reliance on MWRA estimates based on the 2017 census. |
| Reviewer 4: The Discussion reiterates results without deeper interpretation of policy implications. Please expand on how local health managers could operationalize these heat maps (e.g., integrating into existing dashboards, training needs). | We have mentioned that they integrate these systems into existing systems like DHIS2 or LMIS. Secondly, short courses on GIS interpretation and spatial decision making should suffice for using the maps. |
